# DeepBindGCN: Integrating Molecular Vector Representation with Graph Convolutional Neural Networks for Protein–Ligand Interaction Prediction

**DOI:** 10.3390/molecules28124691

**Published:** 2023-06-10

**Authors:** Haiping Zhang, Konda Mani Saravanan, John Z. H. Zhang

**Affiliations:** 1 Shenzhen Institute of Synthetic Biology, Faculty of Synthetic Biology, Shenzhen Institute of Advanced Technology, Chinese Academy of Sciences, Shenzhen 518055, China; 2 Department of Biotechnology, Bharath Institute of Higher Education and Research, Chennai 600073, Tamil Nadu, India; saravananbioinform@bharathuniv.ac.in; 3 School of Chemistry and Molecular Engineering, East China Normal University, Shanghai 200062, China; 4 NYU-ECNU Center for Computational Chemistry at NYU Shanghai, Shanghai 200062, China

**Keywords:** graph convolution network, protein–ligand binding, drug virtual screening, deep learning, DeepBindGCN

## Abstract

The core of large-scale drug virtual screening is to select the binders accurately and efficiently with high affinity from large libraries of small molecules in which non-binders are usually dominant. The binding affinity is significantly influenced by the protein pocket, ligand spatial information, and residue types/atom types. Here, we used the pocket residues or ligand atoms as the nodes and constructed edges with the neighboring information to comprehensively represent the protein pocket or ligand information. Moreover, the model with pre-trained molecular vectors performed better than the one-hot representation. The main advantage of DeepBindGCN is that it is independent of docking conformation, and concisely keeps the spatial information and physical–chemical features. Using TIPE3 and PD-L1 dimer as proof-of-concept examples, we proposed a screening pipeline integrating DeepBindGCN and other methods to identify strong-binding-affinity compounds. It is the first time a non-complex-dependent model has achieved a root mean square error (RMSE) value of 1.4190 and Pearson r value of 0.7584 in the PDBbind v.2016 core set, respectively, thereby showing a comparable prediction power with the state-of-the-art affinity prediction models that rely upon the 3D complex. DeepBindGCN provides a powerful tool to predict the protein–ligand interaction and can be used in many important large-scale virtual screening application scenarios.

## 1. Introduction

Proteins play a key role in most cellular processes; meanwhile, ligands can act as mediators of proteins, and can combat diseases with their physical–chemical properties [1]. However, identifying active compounds experimentally on a large scale is expensive and time-consuming. Hence, computer-aided lead discovery is usually the initial stage of the drug discovery process conducted to reduce the experimental testing burden. Accurately and efficiently predicting the protein–ligand interaction using the computational method is a core component of large-scale drug screening. Deep learning and machine learning have recently been widely applied in drug discovery research [2,3,4,5,6]. With the development of deep learning algorithms and increasing protein–ligand interaction data, especially in the high-resolution atomic structure and experimental binding affinity information, it is possible to apply deep learning to discriminate the binders from non-binders and predict the affinity [7]. Several affinity prediction models have already been developed, such as Pafnucy [8], GraphDTA [9], GAT-Score [10], BAPA [11], and AttentionDTA [12]. Our group also developed DeepBindRG [13] for protein–ligand affinity prediction, with the interface atomic contact information as the input, and DeepBindBC [14] for predicting whether the protein–ligand complexes are native-like by creating a large protein–ligand decoy complex set as a negative training set. Moreover, we developed dense fully connected neural networks (DFCNN) for the preliminary stage of virtual screening since it demonstrates a predictable efficiency [15,16]. Some of our developed models have already been applied in drug candidates and target searching, and show a huge potential in drug development [17,18]. However, several limitations still need attention, both in terms of efficiency and accuracy.

The graph convolutional network (GCN) is a kind of a well-known deep learning tool that can use nodes to contain feature information and edges to contain spatial information between the nodes [19]. GCN has already been well applied to predicting the compound property and the molecular fingerprint [20,21]. Furthermore, the GCN has been successfully used for protein–ligand interaction prediction [9,22]. Wen et al. applied the GCN to predict protein–ligand interactions and achieved promising results in the test set. However, they used the DUD.E as a training dataset and only contained 102 receptors, which is very limited diversity in terms of protein information [22]. This strongly suggests that their model still has ample improvement space. The model’s under-trainings on the protein side can also influence its performance significantly. Thin et al. developed a GCN-based protein–ligand prediction model [9]. It only used GCN for the ligand part, and the protein was represented as a sequence, comparing the pocket with the spatial information. However, this sequence lost spatial information and contained irrelevant information regarding the protein–ligand binding. Furthermore, Moesser et al. integrated protein–ligand contact information in ligand-shaped 3D interaction graphs to improve binding affinity prediction [23]. However, it could only be helpful if the protein–ligand complex was available or was accurately predicted by docking.

It should be noted that many deep learning-based protein–ligand affinity prediction models are rarely used in real applications. Even their RMSE value in the testing set seems to be very small. One major reason attributed to this was that the affinity model was trained over a binding protein–ligand dataset and needs to learn about non-binding. In a real application, the non-binding compounds dominate during screening over a given target. Hence, more than purely developing a deep learning-based affinity prediction model is required to fulfill the requirement of virtual screening. Developing a model trained with binding and non-binding data is needed to identify whether protein–ligand was binding. For instance, we have previously developed DFCNN and DeepBindBC models to identify whether the protein and ligand are binding. These two models have successfully helped to identify a given target’s inhibitors with experimental validation available in our previous work [16,17,18,24,25]. Moreover, combining the protein–ligand binding prediction model with the affinity prediction model can be more powerful in identifying strong affinity candidates. As aforementioned, hybrid screening has been used to virtualize potential drugs for given targets [26]. However, we still lack a model that can screen over a database size of 100,000~1,000,000 accurately and efficiently with the ability to distinguish between the spatial and physical–chemical features of protein–ligand binding.

In our work, we have used a graph to represent the protein pocket and ligand, respectively, and the GCN model with two inputs and one output to fully train over a large protein–ligand PDBbind dataset [27]. The diversified structure database PDBbind guarantees the robustness of model performance. We also evaluated the model performance using the known binding and non-binding data. We also showed its application in drug candidate screening for the target TIPE3 and PD-L1 dimers. Our result reveals that DeepBindGCN can be a valuable tool to rapidly identify reliable, strong-binding protein–ligand pairs, and can be an essential component of a hybrid, large-scale screening pipeline.

## 2. Results

**Figure 1:**
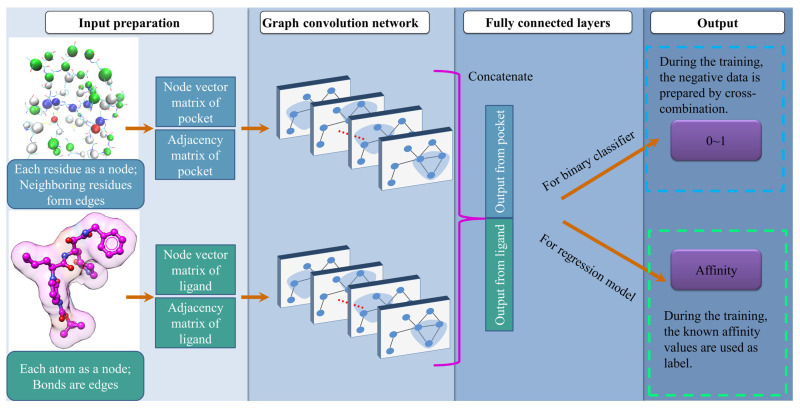
Schematic representation of model construction.

The DeepBindGCN_BC, a binary classifier of binders and non-binders, and DeepBindGCN_RG, a protein–ligand binding affinity predictor workflow, are shown in Figure 1. We observed that the input preparation and model architecture were highly consistent during the training and application, except that one output was 0~1 for binary classification, and the other was the continuous output value for affinity prediction. Several cutoff values of pocket were tried, and we chose the final cutoff that showed the best performance. The DeepBindGCN uses only the protein pocket and ligand information; hence the prediction is independent of protein–ligand complexes. Each residue in the protein pocket was considered as a node, and each neighboring residue pair was taken as an edge to form a graph representation for the protein pocket. Similarly, each atom in the ligand was considered as a node, and each bond was an edge to form a graph representation for the ligands. Since the 1D SMILES (simplified molecular-input line-entry system, a one line notation describing the structure of chemical species using short ASCII strings) notation of the ligand contain the atom type and bond information, the SMILES representation was deemed to be enough for the ligand input. The training and testing data were obtained from the well-known PDBbind dataset. Each pocket and ligand graph representation was sent to the graph convolution network. Later, they concatenated their output by fully connected layers; the final output was used for either the binary classification tasks (DeepBindGCN_BC) or affinity prediction (DeepBindGCN_RG), respectively. The major advantage of this architecture is that there is no explicit conformation searching process; hence, the prediction process would be accelerated and less prone to inaccuracies resulting from incorrect docking conformations.

### 2.1. The Performance of DeepBindGCN_BC and DeepBindGCN_RG on Training and Test Set

The area under the ROC Curve (AUC), true-positive rate (TPR), precision, and accuracy of the training set and test set over the 2000 epoch training for the DeepBindGCN_BC were recorded and shown in Figure S1 and Table S1. The AUC values fell around 0.86~0.87 and 0.84~0.85 after 400 epochs when using the pocket cutoff values of 0.6 nm and 0.8 nm, respectively, indicating that the training has fully converged in epoch 2000. This final pocket cutoff value was selected after testing with several other values. The cutoff value must be sufficient to preserve comprehensive information regarding generalized differences between the crystal-positive representations and negative docking representations, such as covalent bonds between the ligand and protein, but not so small as to lose a significant amount of detailed information. The preservation of unnecessary information causes noise that hinders the model’s capacity to recognize contacts if the cutoff value is too high. Although there was not much of a difference observed in the performance of the model between the two cutoffs, comparing the performance of the 0.6 and 0.8 nm cutoff values helped us to understand how the performance was influenced by the pocket size. Our observations revealed that the DeepBindGCN_BC performed better on the testing set when the pocket cutoff of 0.6 nm was used according to the AUC, TPR, precision, and accuracy performance metrics, as shown in Table S1. For instance, the DeepBindGCN_BC had AUC, TPR, precision, and accuracy values with the cutoff of 0.6 nm at epoch 2000 being 0.8788, 0.6863, 0.6767, and 0.8396, respectively, corresponding to the values of 0.8537, 0.6175, 0.6552, and 0.8231 with the pocket cutoff of 0.8 nm, which thereby indicates a relatively better performance with the pocket cutoff value of 0.6 nm.

The root mean square error (RMSE), mean square error (MSE), Pearson correlation, Spearman correlation, and the concordance index (CI, the larger, the better) of the training set and test set over the 2000 epoch training for the DeepBindGCN_RG are shown in Figure S2 and Table S2. We noted that the RMSE had values around 1.3 and 1.1~1.3 after 400 epochs when using the pocket cutoff values of 0.6 nm and 0.8 nm, respectively, indicating that the training was fully converged. The DeepBindGCN_RG performed better according to the performance metrics RMSE, MSE, Pearson correlation, Spearman correlation, and CI, respectively. For instance, DeepBindGCN_RG with the pocket cutoff of 0.8 nm had RMSE, MSE, Pearson correlation, Spearman correlation, and CI values of 1.2107, 1.4657, 0.7518, 0.7410, and 0.7756 in epoch 2000, respectively, corresponding to the values of 1.3361, 1.7852, 0.7141, 0.7098, and 0.7628 when the pocket cutoff was 0.6 nm, which thereby demonstrates a better performance with the pocket cutoff value of 0.8 nm. It demonstrates that the close-contacting ligand and residue information are sufficient for accurately predicting whether the protein–ligand is indeed binding, whereas long-range contact information sometimes could lead its prediction to be inaccurate [28]. However, long-range pocket residue information is also important to accurately predict how strong the protein–ligand is binding [29]. To accurately estimate the binding affinity, most of the residues that have contributed to the binding should be considered.

### 2.2. The Performance of DeepBindGCN_BC and DeepBindGCN_RG on the DUD.E Dataset

In order to prove that our network can recognize both non-binders and binders correctly, we have compiled an extra testing set from a selection of targets in the PDB database from which the targets of the DUD.E dataset were drawn. The extra testing dataset consisted of decoys and binders of a specific target from the DUD.E dataset. The performances of DeepBindGCN_BC and DeepBindGCN_RG on some DUD.E-containing extra testing datasets are listed in Tables S3 and S4, respectively. DeepBindGCN_BC formed an AUC value above 0.7 for 20 cases from the DUD.E datasets, as shown in Table 1 and Table S3. Some protein–ligand datasets were predicted into all 0 values, which indicated no binding. A possible explanation for this was that the binding pocket we selected could not guarantee exact binding with those ligands. Furthermore, the data may have contained several false-positive experimental results since there were no crystal structures present as a strong proof of binding.

We also assessed the DeepBindGCN_RG on the DUD.E dataset with affinity values, as shown in Table S4. Interestingly, DeepBindGCN_RG performed well over most datasets in terms of the RMSE values. The average RMSE of 102 therapeutic targets-related datasets was 1.1893. We observed that more than 65 protein target-related datasets had a RMSE smaller than 1.2, as shown in Table 2, which was extremely accurate compared to most of the current affinity prediction methods. On the other hand, the Pearson correlation, Spearman correlation, and CI also demonstrated that prediction and experimental outcomes usually have a weak correlation. This is possibly because, for each dataset, many compounds with a different affinity have similar structures, making the model extremely challenging to detect for the difference in binding affinity. The results of our investigation, which involved a thorough analysis of the structure of numerous ligands in the DUD.E dataset, indicate our model’s relative performance. The low RMSE and MSE values can guarantee that the DeepBindGCN_RG can correctly identify strong-affinity binders. The metrics of DeepBindGCN_RG’s performance are better than those of other standard models, but there is still room for improvement. This can be performed by sampling a large amount of negative data and optimizing parameters, both of which take a very long time to then subsequently develop reproducible studies.

### 2.3. Virtual Screening by DeepBindGCN against the TIPE3 and PD-L1 Dimers as Self-Concept-Approve Examples

**Figure 2:**
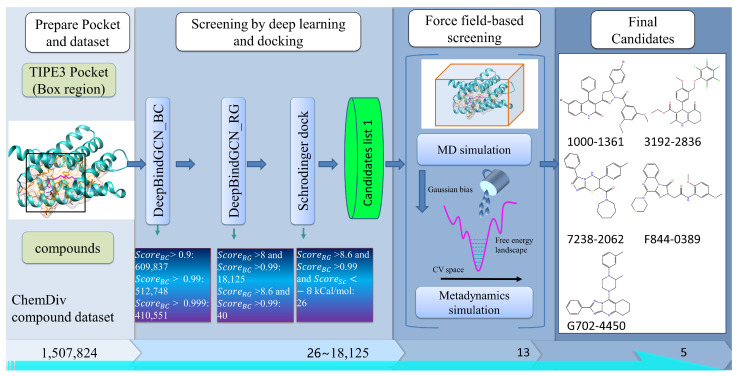
The virtual screening procedure integrates DeepBindGCN models with other methods to identify highly reliable drug candidates for TIPE3.

The TIPE2 (tumor necrosis factor-alpha-induced protein 8-like 2) and programmed cell death ligand 1 (PD-L1) proteins significantly regulate cancer and inflammatory diseases. Knowing its structure and sequence, as well as the essential amino acid residues involved in its ligand binding, sheds light on how it functions while rendering it much easier to discover new anticancer drugs. The ability to explore the inhibitory potential of these proteins on a broad scale has been made feasible by recent developments in deep learning-based drug screening. In light of the aforementioned, we conducted a thorough screening against the proteins TIPE2 and PD-L1. The large-scale virtual screening of small molecular chemicals against the target proteins had been employed, and its methodology is schematically illustrated in Figure 2, which integrates many different methods, including DeepBindGCN, molecular docking, molecular dynamics (MD) simulation, and meta-dynamics-based binding free energy landscape calculation. The MD and meta-dynamics simulation details are described in Supplementary Material Section S2. The MD simulation can provide more detailed and accurate protein–ligand interaction information. However, it is time-consuming and utilizes lots of computational resources. Therefore, it was placed at the end of the screening procedure. In this way, only a few candidates selected by deep learning or docking can be employed with the MD simulation. We developed a similar strategy in our recently published works, and several identified compounds by this strategy have also been proven by the relevant experiments [17,18,25].

In this study, we considered the TIPE3 protein, which is defined as a transfer protein for secondary lipid messengers that is upregulated in human lung cancer tissues [30]. Recent research have revealed its important role in cancer proliferation, which is believed to be a novel cancer therapeutic target [31]. However, there are still no effective compounds that can inhibit its function. In this work, we obtained 40 candidate compounds with DeepBindGCN, as shown in Table 3. The calculated pK_a_ (DeepBindGCN_RG) typically resulted in a positive value when the K_i_, K_d_, or IC_50_ measures were less than 1 M. We also docked these candidates with TIPE3 using Schrödinger software to obtain the potential binding conformations. The docking score is listed in Table 3.

**Figure 3:**
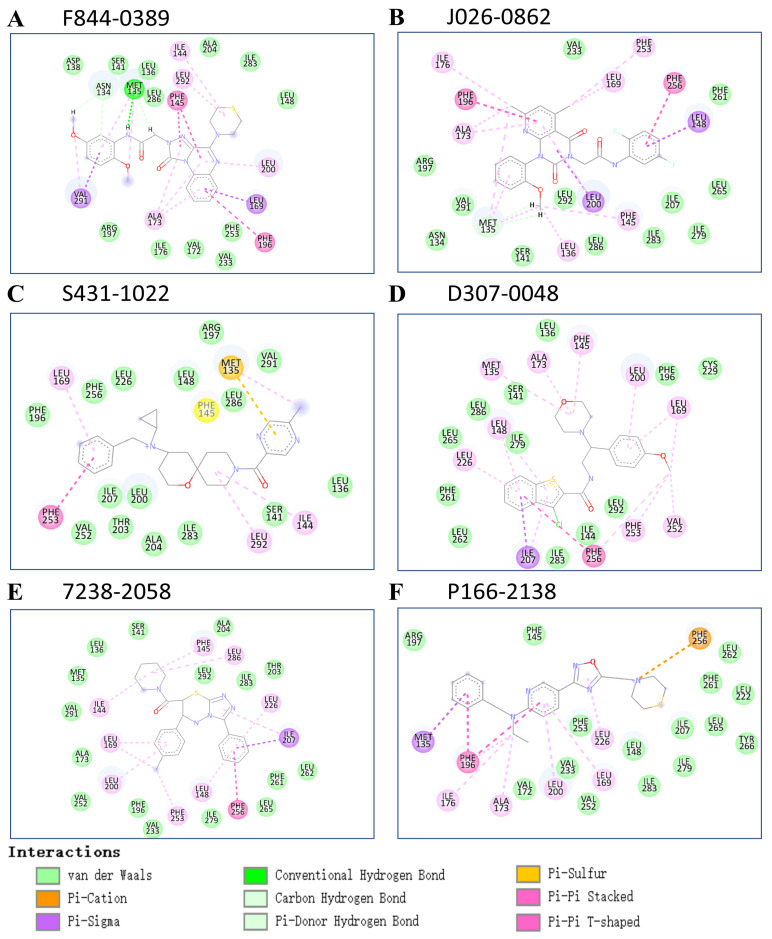
Two-dimensional ligand interaction diagram of TIPE3-F844-0389 (**A**), TIPE3-J926-0862 (**B**), TIPE3-S431-1022 (**C**), TIPE3-D307-0048 (**D**), TIPE3-7238-2058 (**E**), and TIPE3-P166-2138 (**F**), respectively, resulted from docking calculations were presented. Green balls represent hydrophobic residues, whereas dark blue balls represent charged residues. These interaction diagrams have been plotted using Discovery Studio Visualizer.

For the ease of analysis, we have clustered the candidate’s list into six groups, and the structures of cluster center compounds are shown in Figure S3. We observed that clusters 1 and 2 had the largest number of group members. Notably, the cluster center structure contained several benzene-like substructures, indicating that pi-related interactions may be necessary for the strong binding with TIPE3. We also found that the representative structures of clusters 1, 2, 3, 4, and 5 have a linear shape, respectively, indicating that the linear shape molecules may easily enter the binding cavity and achieve tight binding. Furthermore, all the representative structures were relatively flat, which may therefore facilitate the entering into the binding pocket more easily. To further explore the predicted TIPE3′s interaction details with the representative structures, we have plotted its 2D pocket–ligand interaction details, as shown in Figure 3. Consistent with our previous assumption, we observed that most interactions were strongly maintained by Pi-related interactions. While there were many pi-related interactions for most of these compounds with TIPE3, only F844-0389 was found to have formed one hydrogen bond with TIPE3, indicating the hydrogen bond may not be the dominant force for tight TIPE3 binding.

In the post-genomics era, immunotherapies targeting programmed cell death ligand 1 (PD-L1) have recently raised a lot of optimism for cancer patients. Successful anti-PD-L1 treatment involves renewing worn-out T cells and eradicating immunosuppressive cancer cells. Compound-induced dimerization of PD-L1 is an effective way to prevent PD-L1-PD-1 binding, leading to the inhibition of cancer cell proliferation. Considering the above, we have performed DeepBindGCN screening over the compound database against PD-L1. The compounds with DeepBindGCN_RG > 8.6 were selected as candidates, as shown in Table S5.

We obtained six representative structures by clustering the candidates into six groups, as shown in Figure S4, with cluster 5 having the largest group members. The representative structures of clusters 1, 2, and 3 have similar shapes, while the representative clusters 3, 4, and 5 share similar linear shapes. Interestingly, except for representative structure 2, all five representative structures were compounds containing the pentacyclic ring. The 2D interaction of the predicted representative compounds with the PD-L1 dimer from Schrödinger docking was shown in Figure S5. Most compounds interacted with the PD-L1 pocket, including through hydrogen bonds, Pi-related interactions, salt-bridge interactions, etc. It should be noted that Schrödinger still needs to dock K305-0238 and E955-0720 into the selected PD-L1 pocket successfully.

**Figure 4:**
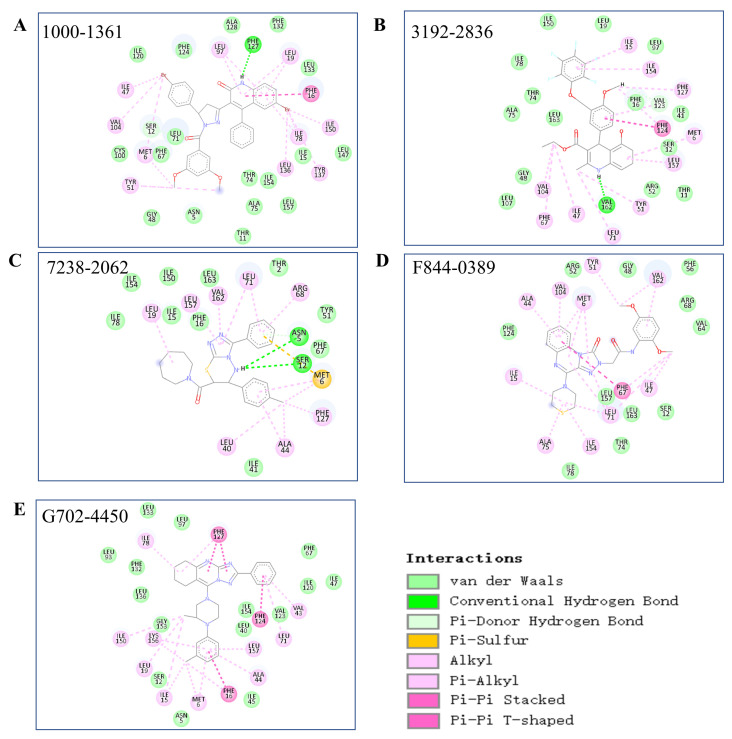
The two dimensional TIPE3 chemical interaction details with the candidate compounds for the last frame from the MD simulation. The protein-ligand two dimensional interaction details of (**A**) TIPE3-1000-1361, (**B**) TIPE3-3192-2836, (**C**) TIPE3-7238-2068, (**D**) TIPE3-F844-0389 and (**E**) TIPE3-G702-4450 are presented.

We further carried out MD and meta-dynamics simulations to check the binding stability of the predicted protein–ligand pairs. The candidates that showed favorable binding with the three targets according to the free energy landscape from the meta-dynamics simulation were selected for further analysis, as shown in Figure S6. We noticed that except for F844-0389 (with a RMSD value around 0.3~0.5), the calculated RMSD value of these selected candidates for the TIPE3 had very small values (around 0.1~0.3 nm) and a low fluctuation, as shown in Figure S7, indicating that these candidates have a very stable binding ability. The protein-compound interaction details of the last frame from the MD simulation are shown in Figure 4.

**Figure 5:**
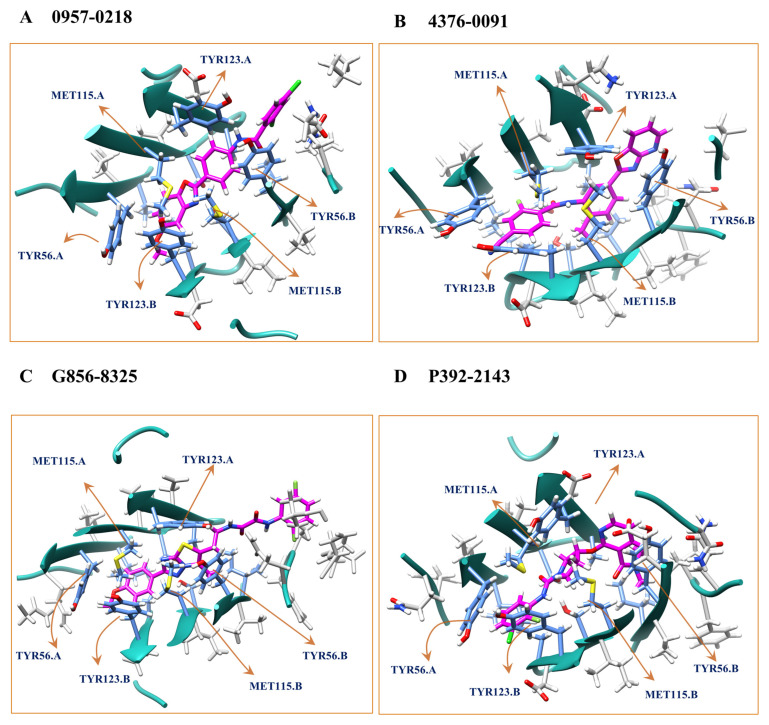
Three-dimensional view of PD-L1-representative cluster center compounds and two-dimensional ligand interaction diagram of PD-L1-0957-0218 (**A**), PD-L1-4376-0091 (**B**), PD-L1-G856-8325 (**C**), and PD-L1-P392-2143 (**D**) respectively, resulted from docking calculations were presented. Green balls represent hydrophobic residues, whereas dark blue balls represent charged residues. The interaction diagrams have been plotted using Discovery Studio Visualizer.

The root mean square deviation (RMSD) of the selected candidates during the MD simulation for the PD-L1 dimer is shown in Figure S8. Notably, the RMSD values of 4376-0091 and P392-2143 had very small values (of around 0.1~0.2 nm), indicating that their binding was stable. The interaction details of the selected candidates with the PD-L1 dimer are shown in Figure 5, and the detailed 2D interactions are shown in Figure S9. We observed that the binding pocket contains many non-polar residues, and the interaction between the PD-L1 dimer with the compounds was dominant with hydrophobic interactions. This indicates that the compounds may act as a molecular glue to promote and stabilize the PD-L1 dimerization. MD simulations can often be difficult to converge within 40 ns or even much longer periods (e.g., 200 ns) for certain circumstances, therefore the RMSD in MD simulations are typically insufficient to assess whether a ligand binds well or not. This is the for which we require meta-dynamics simulations. For example, 4376-0091 had a constant RMSD value for 40 ns, but additional meta-dynamics suggested that it favored the unbound area, thereby indicating that 4376-0091 may not be a viable choice for further stage assessment.

## 3. Discussion

The proposed GCN-based model is more efficient than traditional docking and deep learning-based methods. Since it does not depend on the protein–ligand complex, it can save time and resources to pre-process the input by docking. In many other complex structure-based models, most of the time is spent exploring binding conformation, and the prediction would only be reliable if the binding conformations were correct. Using the pre-trained molecular vector to represent the residues, the GCN-based model displayed an obvious improvement, indicating that our model can identify physical–chemical features and spatial information. The model’s performance on the DUD.E dataset was also good, indicating it is highly advantageous for real applications. This model has a great potential as a core component of large-scale virtual screening. The method strongly complements many existing methods, such as docking, MD simulation, and other deep learning methods; hence, it can easily be integrated into a hybrid screening strategy. The methods can also be used to screen de novo compounds by combining them with molecular generative models, similar to our previous work [5].

We assessed its running time in the virtual screening process to check its efficiency. With the CUDA acceleration, we found that DeepBindGCN_BC and DeepBindGCN_RG spent about 45.5 s and 22.2 s to complete the prediction of 50,000 protein–ligand pairs, respectively, with an Intel CPU core (2.00 GHz) and a GeForce RTX 2080 Ti GPU card. With only CPU, it took about 57.8 s and 61.9 s for DeepBindGCN_BC and DeepBindGCN_RG to finish the prediction of 50,000 protein–ligand pairs, respectively, with 40 Intel CPU cores (2.00 GHz). This indicates that DeepBindGCN_BC or DeepBindGCN_RG only need 0.0004~0.0012 s to complete a prediction, which is at least ten thousand times faster than traditional docking (which usually takes tens of seconds to several minutes) or docking-dependent deep learning-based protein–ligand affinity prediction methods. In summary, large-scale virtual screening would greatly benefit from DeepBindGCN’s efficiency.

To compare the performance of the DeepBindGCN_RG-like model with other affinity prediction models on the PDBbind core set, we trained a DeepBindGCN_RG_x model over datasets without the PDBbind core set 2013 and 2016 (CASF-2016) [32]. The training details are provided in Supplementary Material Section S1. The performance of DeepBindGCN_RG_x with different epochs on the PDBbind core sets 2013 and 2016 (CASF-2016) are shown in Tables S6 and S7. Similar to DeepBindGCN_RG, we considered a model with a 2000 epoch as the final model. Since many other protein–ligand affinity prediction models have been widely evaluated on these two datasets, we collected other method performances from the relevant literature reports and listed them in Table 4. The methods used for comparison include KDEEP [33], Pafnucy [8], midlevel fusion [34], GraphBAR [35], AK-score-ensemble [36], DeepAtom [37], PointNet(B) [38], PointTransform(B) [38], AEScore [39], ResAtom-Score [40], DEELIG [41], PIGNet (ensemble) [42], BAPA [11], SE-OnionNet [43], and DeepBindRG [13]. We observed that our DeepBindGCN_RG_x exhibited a comparable performance with most of the state-of-art models. We noted that some methods had a better RMSE or R-value than our DeepBindGCN_RG_x, but they all utilized the interface information of the crystal structure of the protein–ligand complex. Moreover, only our method in Table 4 was found to be independent of the protein–ligand complex, while others depended on the experimental complex.

As the experimental complex is unavailable in a real application, and the protein–ligand complex is typically obtained by molecular docking, the method will therefore perform poorly in such a scenario due to unreliable docking conformation. However, our method’s performance is independent of the protein–ligand complex, and its performance therefore would be stable in such a real application. Its good performance in the DUD.E dataset also strongly supports this assumption. It is the first time a deep learning-based model has achieved an RMSE value of 1.4190 and Pearson r value of 0.7584 in the PDBbind v.2016 core set, respectively, without any experimental protein–ligand complex. This affinity prediction model is valuable in various real-case virtual screening applications. In contrast, most current affinity prediction models are rarely used in real applications. Notably, according to previous research [44], the scoring of most of the traditional docking methods needs to be better with the PDBbind v.2016 core set (CASF-2016); most have a Pearson correlation coefficient below 0.6. Interestingly, only ΔVinaRF20 [45] has achieved a Pearson correlation coefficient of ~0.75, which also depended on accurate complex structures.

To explore whether the vector representation of amino acids performed better than the one-hot representation, we trained a model with one-hot representation with the same model architecture, training, and validation set. Figure S10, and Tables S6 and S7 show the performance over validation with different epochs. We observed that the model’s performance was better than DeepBindGCN. Similar to the DFCNN [15,16], the DeepBindGCN can be applied quickly and accurately to identify the potential protein target. The DeepBindGCN has inherited the efficiency of the DFCNN model, which is also independent of the protein–ligand docking structure. In the meantime, the DeepBindGCN was much more efficient in keeping the spatial information within the ligands and pockets through graph representation. Given the importance of spatial information in many protein–ligand interactions, inverse target searching using the DeepBindGCN should be more effective in identifying targets for specific molecules.

It is worth noting that the 40 ns MD and 40 ns meta-dynamics simulations are not always sufficient for fully validating the stability of a binding pose and binding free energy landscape in some cases. The meta-dynamics free energy landscape was used here to estimate the binding preference. Longer simulations should provide more reliable results and can even calculate the binding free energy if the meta-dynamics simulation has fully converged after a long simulation, but sometimes this is difficult since the collective variable (CV) we chose may not be suitable for the binding free energy calculation. We used a 40 ns MD simulation and meta-dynamics to consider the balance between the efficiency and accuracy during screening applications. We strongly recommend that the user carry longer simulation times (for instance, 100–300 ns) if enough computational resources are available. Previously, we ran a 100 ns MD simulation and 100 ns meta-dynamics as the final screening step to successfully identify an active inhibitor for TIPE2 [17]. In our previous work, we also performed similar extended MD simulations and calculated the free energy [18]. We also should keep in mind that the force field itself contained inaccuracies [18]. It is imperative that the force field potential utilized for the evaluation of the interaction between the ligand and protein incorporates the impact of solvent polarization effects [46]. When taking into account the inadequate sampling of the conformations and the varying protonation states of both the ligand and binding site residues, it becomes apparent for why the docking calculations are unsuccessful in accurately predicting the (de)solvation energy of the ligand and protein, as well as the enthalpy contribution of water molecules to ligand–protein binding [47,48,49,50].

Hence, if the simulation is very long, these inaccuracies may accumulate and lead to inaccurate predictions. Moreover, we found that the CV we chose (coordinate number) was suitable for the fast estimation of the energy that was needed for the ligand to escape the pocket (the tightness of binding), but it would be hard to estimate the exact binding free energy since once the ligand fully solvates (no contact with the pocket), it would need to deposit an abundance of energy to make the ligand go back to the pocket, which makes the exact free energy prediction a very challenging task. More reasonable CVs should be chosen to estimate the binding free energy. Using the coordinate number as a CV with 40–100 ns meta-dynamics simulation should be much more accurate than flexible docking in estimating the binding preference, and hence would be suitable for such screening tasks. The estimation of binding free energy becomes significantly challenging when dealing with the conventional meta-dynamics approach. Therefore, in order to estimate the binding free energy, more efficient and accurate funnel meta-dynamics have been proposed [46]. The discovery of a funnel meta-dynamics approach that is better suited for calculating the protein–ligand binding free energy is both exciting and appreciated.

There is still room for improvement of the model in the future. For instance, we can assess other model architectures, such as automated transit networks (ATN) instead of GCN. Furthermore, we can apply molecular vectors in compounds as well. In this work, the molecular vector for the amino acids has chosen a dimension of 30 for saving disk memory, but in the future, we can try a larger dimension to keep more detailed physical–chemical information and hope to achieve a better performance as a result. The model could also be improved by replacing the fully connected layers with deeper, densely connected layers, or by adding attentional layers. For instance, each chemical group was represented as a node with its molecular vector, and the edge was defined as chemical group neighbors. Also, we can add compound molecular vectors as an independent input. Moreover we can integrate further protein–ligand interaction pair information as the graph input, just as Moesser et al. reported [23]. Furthermore the negative data for the DeepBindGCN_BC model can be further amplified by cross-combining more times, which may have a closer distribution with the application scenario, where many more compounds are inactive. Moreover, a similar strategy can be applied to protein–protein or protein–peptide interaction predictions. The protein interaction interface can be represented by graph representation in a very similar way. Hence, our work can provide s helpful insight into the protein–protein interaction or protein–peptide interaction prediction.

## 4. Materials and Methods

### 4.1. Data Preparation

The training data was downloaded from PDBbind2019 database [51]. The protein pocket size was defined as a cutoff value within the known ligand (any atom in the residue within the cutoff value of the ligand will keep the residue as the pocket residue). We tested the cutoff values of 0.6 nm and 0.8 nm in this work. The ligands were represented as molecular graphs by converting the SMILES code to its corresponding molecular graph and extracting atomic features using the open-source chemical informatics software RDKit [52]. The pocket was represented as a graph by defining the residues as nodes and the contacting residue pairs as edges (the cutoff was set as 0.5 nm). We have assessed one-hot and molecular vector representations for the node residue, respectively. A pre-trained mol2vec model generated the molecular vector of the chemical groups with a dimension of 30 (notably, the original mol2vec has a dimension of 300) [53]. We chose a dimension of 30 for less consumption of disk memory, but we encourage testing larger dimensions in the future. The data to pre-train the mol2vec was obtained from 9,216,175 onstock compounds from the ZINC 15 database [54]. Each standard amino acid vector was represented by adding its chimerical group vector. For the small molecule, each atom was denoted as a node with a feature vector representation containing five pieces of information, the same as the GraphDTA [9]. The five pieces of information included the atom symbol (one hot representation), the number of adjacent atoms, the number of adjacent hydrogens, the implicit value of the atom, and whether the atom was in an aromatic structure.

### 4.2. The Dataset for a Binary Classification Task

Through cross-combination, we obtained 52,200 protein–ligand pairs as a negative dataset and divided them into 45,000 as negative training data and 7200 as testing negative data, respectively. From the PDBbind2019 dataset, we obtained 17,400 protein–ligand as positive data, 15,000 as training positive data, and 2400 as testing data, respectively. We ensured that the protein pocket-ligand pairs used in the test set were not in the training set. During the training, the positive training and testing data were used three times to keep the positive and negative data balanced.

### 4.3. The Dataset for the Affinity Prediction Task

We obtained 16,956 protein–ligand datasets with affinity from PDBbind2019 and divided them into 15,000 training and 1956 test datasets, respectively. In the PDBbind2019 dataset, the binding affinities of protein–ligand complexes were provided with K_i_, K_d_, and IC_50_. We transformed the binding affinities into pK_a_ using the following equation:

(1) $$pK_{a}=-{log}_{10}K_{x}$$

where $K_{x}$ represents IC_50_, $K_{i}$, or $K_{d},$ respectively.

The pK_a_ has no unit, but can be converted into energy by following formula:

ΔG = RT ln 10^(pK_a_)

where RT = 0.593 kcal⋅mol^−1^ at the temperature of 298 K.

In such a way, the pK_a_ can be converted into energy with the unit of kcal/mol^−1^. Presently, several researchers have combined the affinity prediction task instead of constructing distinct models for predicting the K_i_, K_d_, and IC_50_ values using discrete data [55,56]. We have adopted their approach in this study [56]. Integrating the K_i_, K_d_, and IC50 data offers the benefit of augmenting the dataset size, potentially enhancing the efficacy of the model’s training. In the future, it is recommended that researchers should develop models to independently predict the K_i_, K_d_, or IC_50_ values using independent training data as the amount of data continues to accumulate significantly. The K_i_ data was exclusively utilized for training the model to predict the pK_a_ based on the K_i_ value, while the K_d_ data was solely employed for training the model to predict the pK_a_ based on the K_d_ value. Similarly, the IC_50_ data was exclusively utilized for training the model to predict the pK_a_ based on the IC_50_ value.

### 4.4. Pre-Train 30-Dimension Molecular Vector to Represent Residues in Pocket

We downloaded 9,216,175 stock compounds from the ZINC15 database as a training dataset, and mol2vec was used for the training. We finally obtained a model that can generate a vector for each given chemical group, and here we set the vector dimension to 30. The obtained model was used to generate the vector of the 20 residues by adding the chemical group vectors within each residue.

### 4.5. Model Construction

**Figure 6:**
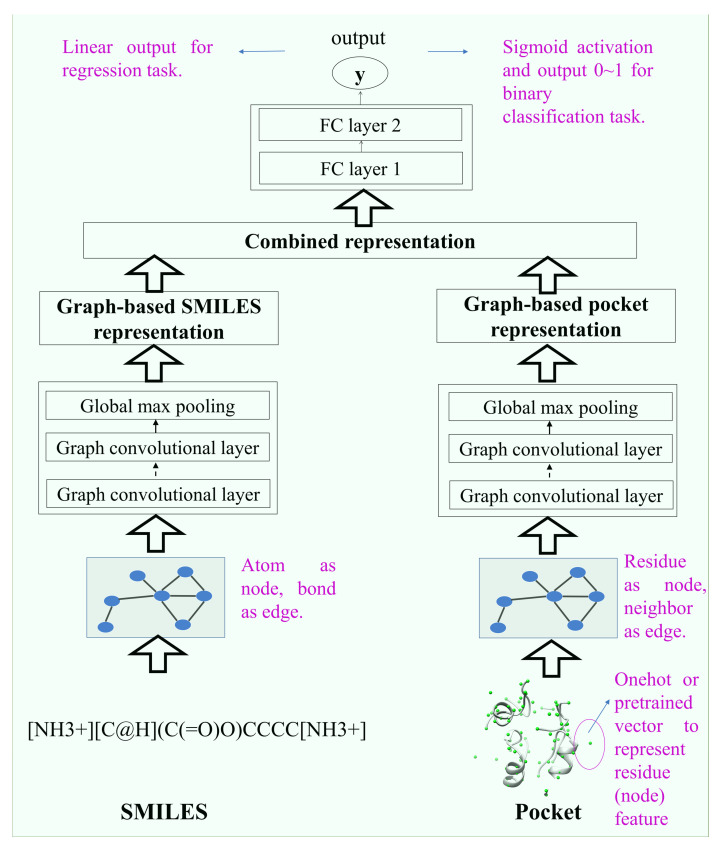
The architecture of the DeepBindGCN model.

The model structure is shown in Figure 6. It consists of two inputs (drug–target pairs) and one output structure. The ligand and pocket graph information flow into the two layers of the graph network. Then, the output of the two graph networks merged into fully connected layers, resulting in the final output of one node. The binary prediction used the sigmoid activation function, which gave a value range of 0~1; for the affinity prediction, the output used linear activation, defined as a continuous measurement of binding affinity for that pair.

### 4.6. Model Training

The torch_geometric module created the input data and constructed the graph neural network. The input data was saved in PyTorch, InMemoryDataset format. The PyTorch was used to perform the training. The number of epochs that we finally chose was based on the performance convergences on the test set.

### 4.7. Model Performance Compared with Other Methods on the DUD.E Dataset

We have downloaded 102 therapeutic-related proteins and their corresponding active and inactive compounds from the DUD.E dataset [57]. We obtained the known active and known inactive DUD.E dataset from the following webpage (https://dude.docking.org/subsets/all, accessed on 15 November 2022). There are known active compounds, known inactive compounds, and decoy compounds in the database, but in this work, we only considered the known active compounds (actives_nM_combined.ism) and inactive compounds (inactives_nM_combined.ism) as an extra test set for DeepBindGCN_BC, and we only considered the known active compounds (actives_nM_combined.ism) and its binding affinity as an extra test for DeepBindGCN_RG. These data were processed into the input format and used as an extra testing set to examine our model performance. The performance matrix AUC, MCC, accuracy, precision, and TPR were used to validate the BC model, while the RMSE, MSE, Pearson correlation, Spearman correlation, and concordance index (CI) were used to validate the RG model. We used the NumPy corrcoef function to compute the Pearson correlation, and the stats.spearmanr function from scipy to calculate the Spearman correlation, respectively.

### 4.8. Virtual Screening of Candidates against Two Targets (TIPE3 and the PD-L1 Dimer)

The atomic coordinates of TIPE3 were retrieved from PDB with the ID 4Q9V [30]. The TIPE3–ligand complex was modeled by the cofactor method in the https://zhanggroup.org/COFACTOR/web server accessed on 15 November 2022 [58]. The PD-L1 dimer was retrieved from PDB with the ID 5N2D [59]. These PDB structures already contained ligands. The pocket was extracted as 0.8 nm from the predicted or known ligands. The dataset Chemdiv with the size of 1,507,824 compounds was used as a virtual screening dataset.

### 4.9. Tools Used in the Analysis

The USCF Chimera, VMD, Schrödinger, pymol, and Discovery Studio Visualizer 2019 were used to generate the structure and to visualize the 2D protein–ligand interactions [60,61,62]. Clusfps (https://github.com/kaiwang0112006/clusfps, accessed on 15 November 2022), which depends on the RDKit [52], was used to cluster the drugs in the dataset. The drug fingerprint was used as an input, with the Ward’s hierarchical agglomerative clustering method used for clustering the candidates into six groups [63,64].

## 5. Conclusions

We have developed DeepBindGCN_BC to identify accurate protein–ligand binding and DeepBindGCN_RG to further estimate the protein–ligand binding affinity. Our GCN-based model not only helps to identify binding ligands, but also helps to identify strong binding ligands, which are often more likely to be developed into drugs. These models have used the graph convolution network to represent spatial information more efficiently. Furthermore, we have added the molecular vector representation to enhance the pocket physical–chemical feature. Moreover, we have assessed the model in a much diversified DUD.E dataset and achieved a good performance, indicating the reliability and practicality of our method. Also, to demonstrate its application in virtual screening, we have developed a pipeline and screened it over two cancer-related therapeutic targets, TIPE3 and the PD-L1 dimer, as proof-of-concept applications. We also highlight its potential in other tasks, such as inverse target screening, specificity calculation, and iteratively screening de novo compounds by integrating with molecule generative models. We have deposited the source codes of our model on GitHub for the user’s convenience. The models and the screening pipeline presented here would greatly facilitate computer-aided drug development.

## Figures and Tables

**Table 1:** The performance of DeepBindGCN_BC on some of the DUD.E datasets with AUC values larger than 0.7. PDBID—protein Data bank Identifier; AUC—area under the ROC curve; TPR—true-positive rate; MCC—Matthews correlation coefficient; data_size—number of entries in the dataset; pos_size—number of binders in the dataset; and neg_size—number of non-binders in the dataset.

PDBID	AUC	TPR	Precision	Accuracy	MCC	Data_Size	Pos_Size	Neg_Size
3BWM	1	0.8537	1	0.8571	0.3492	42	41	1
3KRJ	0.9378	0.7558	1	0.7589	0.1944	394	389	5
2FSZ	0.8597	0.9173	0.9661	0.8948	0.4686	1492	1366	126
1XL2	0.8517	0.4639	0.9887	0.491	0.1817	1607	1511	96
3D0E	0.8424	0.6498	0.9809	0.6692	0.3015	260	237	23
2NNQ	0.8364	0.9362	0.8	0.7778	0.3251	63	47	16
3L5D	0.8266	0.9133	0.9665	0.8892	0.3445	641	600	41
2RGP	0.819	0.7463	0.9322	0.7538	0.4423	2027	1620	407
2HZI	0.8138	0.6895	0.94	0.7059	0.366	493	409	84
3G0E	0.8057	0.6887	0.9924	0.6899	0.1338	387	379	8
3L3M	0.8029	0.5301	1	0.5355	0.1125	1057	1045	12
1SJ0	0.8025	0.7057	0.9617	0.7078	0.2678	1451	1315	136
3F07	0.7875	0.8307	0.9298	0.8112	0.4865	392	319	73
3CCW	0.7652	0.5878	0.9969	0.592	0.1124	549	541	8
1UDT	0.7414	0.7536	0.9531	0.7413	0.231	1063	970	93
2CNK	0.735	0.1928	0.9891	0.2495	0.1118	509	472	37
3ODU	0.7184	0.8372	0.8372	0.7544	0.3372	57	43	14
3D4Q	0.7178	0.8202	0.9524	0.7971	0.2392	345	317	28
2AYW	0.7089	0.2182	0.9638	0.2946	0.1154	1093	976	117
2AA2	0.7052	0.2217	1	0.229	0.0515	214	212	2

**Table 2:** The performance of DeepBindGCN_RG on some DUD.E datasets with RMSE values smaller than 1. PDBID—protein data bank identifier; RMSE—root mean square error; MSE—mean square error; CI—concordance index; and data_size—number of entries in the dataset.

PDBID	RMSE	MSE	Pearson	Spearman	CI	Data_Size
3BIZ	0.6866	0.4714	0.1794	0.1800	0.5570	221
2AZR	0.7134	0.5089	0.2293	0.2654	0.5903	284
1UYG	0.7880	0.6209	0.3155	0.2981	0.6089	88
3M2W	0.7958	0.6334	0.3754	0.3063	0.6073	184
3EQH	0.8114	0.6584	0.3547	0.3277	0.6159	308
2ETR	0.8119	0.6592	0.2780	0.2687	0.5961	219
3F9M	0.8177	0.6686	0.1705	0.1740	0.5611	144
1KVO	0.8184	0.6697	0.1789	0.1481	0.5510	176
1SQT	0.8194	0.6715	0.2473	0.2282	0.5777	375
3D0E	0.8439	0.7122	0.2704	0.2272	0.5797	237
3L5D	0.8480	0.7191	0.3180	0.3432	0.6187	600
1LRU	0.8956	0.8021	0.2213	0.2362	0.5805	173
3NF7	0.9010	0.8119	0.1790	0.1021	0.5353	185
3HMM	0.9035	0.8163	0.0380	0.0055	0.5010	235
2ICA	0.9056	0.8201	0.3269	0.3630	0.6210	324
2HZI	0.9088	0.8258	0.5412	0.5701	0.6958	409
3KGC	0.9121	0.8319	−0.0222	0.0049	0.5013	488
2HV5	0.9258	0.8572	0.0512	0.0530	0.5178	606
3EL8	0.9303	0.8654	0.2629	0.2570	0.5875	1271
2OJG	0.9386	0.8810	0.5505	0.5713	0.7045	81
1D3G	0.9397	0.8831	0.0503	0.0742	0.5269	227
1BCD	0.9496	0.9017	0.3138	0.2846	0.5974	1976
2V3F	0.9621	0.9256	0.3420	0.2885	0.5987	55
3CCW	0.9665	0.9341	0.2556	0.2955	0.6004	541
2QD9	0.9730	0.9468	0.3492	0.3509	0.6196	2218
3KRJ	0.9770	0.9545	0.2654	0.2395	0.5826	389
3CQW	0.9779	0.9562	0.2804	0.2742	0.5933	588
2ZNP	0.9779	0.9564	0.1656	0.1517	0.5510	713
2OF2	0.9816	0.9635	0.2678	0.2355	0.5797	919
830C	0.9833	0.9668	0.2000	0.1883	0.5641	1644
3LAN	0.9854	0.9709	0.1809	0.1732	0.5596	1201
2OJ9	0.9918	0.9836	0.4426	0.4041	0.6388	373
3MAX	0.9936	0.9873	0.0286	0.0379	0.5130	413
1J4H	0.9965	0.9930	−0.1850	−0.1821	0.4383	165
3G0E	0.9967	0.9935	0.0037	−0.0001	0.4966	379
1UDT	0.9988	0.9976	0.4255	0.4115	0.6419	970

**Table 3:** The top predicted candidates from DeepBindGCN_BC and DeepBindGCN_RG for the TIPE3 protein.

Compound ID	DeepBindGCN_BC	DeepBindGCN_RG	Schrödinger Score
G858-0261	1.0000	9.0349	−9.5265
D491-8162	1.0000	9.0312	−7.7093
D307-0048	1.0000	9.0666	−8.1571
3192-2836	1.0000	9.0383	−9.2614
1000-1361	1.0000	9.0062	−11.0240
8014-2686	1.0000	9.0927	−7.5773
S049-0833	1.0000	9.1489	−8.6633
V010-1363	1.0000	9.0040	−8.4298
F844-0391	1.0000	9.0815	−7.3199
S556-0709	1.0000	9.0541	−7.0894
C200-4178	1.0000	9.0407	−7.6719
F844-0420	1.0000	9.4370	−8.2764
J026-0862	1.0000	9.0249	−8.6472
C258-0578	1.0000	9.0228	−8.3843
C200-0812	0.9999	9.0793	−9.2365
S561-0589	0.9999	9.0254	−8.1083
P166-2237	0.9999	9.6668	−8.7043
V006-0149	0.9999	9.0806	−8.3682
P074-3068	0.9999	9.0822	−9.0598
7238-2062	0.9999	9.0083	−8.6726
G702-4450	0.9998	9.0540	−9.5383
Y031-6037	0.9998	9.0993	−7.3331
L827-0130	0.9998	9.0523	−8.5650
F844-0390	0.9998	9.2186	−7.7939
K305-0239	0.9997	9.0028	None
7238-2058	0.9995	9.0692	−8.5960
P166-2138	0.9994	9.7564	−8.4074
8131-1510	0.9993	9.0366	−8.5564
S543-0517	0.9992	9.3285	−7.5612
F844-0389	0.9992	9.3423	−8.7665
L824-0015	0.9990	9.3347	−7.1463
G702-4471	0.9986	9.0317	−8.7210
P074-3101	0.9985	9.0468	−8.5187
Y043-1747	0.9980	9.0451	−7.2643
V008-1643	0.9972	9.0701	None
8015-5821	0.9964	9.0231	−9.7178
S431-1022	0.9954	9.3035	−8.2101
S591-0082	0.9952	9.0663	−6.7099
P166-2131	0.9944	9.3489	−6.5523
C301-8688	0.9939	9.3810	−8.3378

**Table 4:** Performance comparison of our DeepBindGCN_RG_x method with other methods in predicting the experimental affinity on the PDBbind v.2016 core set (CASF-2016 core set) and v.2013 core set.

Test Set	Methods	RMSE	Pearson R	Spearman R
PDBbind v.2016 core set	DeepBindGCN_RG_x	1.41	0.75	0.743
	KDEEP	1.27	0.82	
	Pafnucy	1.42	0.78	
	midlevel fusion	1.30	0.81	0.807
	GraphBAR(dataset 4, Adj-2)	1.41	0.77	
	AK-score-ensemble	1.29		
	DeepAtom	1.23	0.83	
	PointNet(B)	1.26	0.83	0.827
	PointTransform(B)	1.19	0.85	0.853
	AEScore	1.22	0.83	
	ResAtom-Score		0.83	
	DEELIG		0.88	
	PIGNet (ensemble)		0.76	
	BAPA	1.30		
PDBbind v.2013 core set	DeepBindGCN_RG_x	1.49	0.74	0.727
	SE-OnionNet	1.69	0.81	
	DeepBindRG	1.81	0.63	
	DEELIG		0.89	
	GraphBAR(dataset 4, best)	1.63	0.70	
	BAPA	1.45		

## Data Availability

The proposed models and the scripts are available in GitHub public repositories (https://github.com/haiping1010/DeepBindGCN, accessed on 15 November 2022).

## Supplementary Materials

The following supporting information can be downloaded at: https://www.mdpi.com/article/10.3390/molecules28124691/s1, Supplementary methods section: Figure S1: The DeepBindGCN_BC performance over the 2000 epoch training; Figure S2: The DeepBindGCN_RG performance over the 2000 epoch training; Figure S3: The six clusters and corresponding representative cluster center structure of top predicted candidates from DeepBindGCN_BC and DeepBindGCN_RG for the TIPE3; Figure S4: The six clusters and corresponding representative cluster center structure of top predicted candidates from DeepBindGCN_BC and DeepBindGCN_RG for the PD-L1; Figure S5: The snapshot and 2D plot of PD-L1 with representative cluster center compounds from docking; Figure S6: The calculated free energy landscape from metadynamics simulation for those candidates that have favorable binding with the given target; Figure S7: The RMSD value of selected compounds with the TIPE3 during the MD simulation; Figure S8: The RMSD value of selected compounds with the PD-L1 during the MD simulation; Figure S9: The 2D interaction snapshot of PD-L1 dimer with candidate compounds for the last frame from the MD simulation; Figure S10: The performance of binary classification and affinity prediction models with onehot like residue representation over the 2000 epoch training; Table S1: Performance of DeepBindGCN_BC on the test set during the training with epoch interval 100; Table S2: Performance of DeepBindGCN_RG on test set during the training with epoch interval 100; Table S3: The performance of DeepBindGCN_BC (pocket cutoff 0.6 nm) on the DUD.E dataset; Table S4: The performance of DeepBindGCN_RG (pocket cutoff 0.8 nm) on the DUD.E dataset; Table S5: The top predicted candidates from DeepBindGCN_BC and DeepBindGCN_RG for the PD-L1 dimer; Table S6: The performance of DeepBindGCN_RG_x with different training epoch on the PDBbind v.2016 core set (CASF-2016); Table S7: The performance of DeepBindGCN_RG_x with different training epoch on the PDBbind v.2013 core set; Table S8: Performance of DeepBindGCN_BC_onehot representation on test set during the training with epoch interval 100; Table S9: Performance of DeepBindGCN_RG_onehot on test set during the training with epoch interval 100. References [18,65,66,67,68,69,70,71,72,73,74,75,76,77,78] are cited in the supplementary materials.
