# Supplementary material for: DeepBindGCN: Integrating Molecular Vector Representation with Graph Convolutional Neural Networks for Protein–Ligand Interaction Prediction
Source: Molecules. 2023 Jun 10;28(12):4691. doi: 10.3390/molecules28124691 (PMC10301867; doi:10.3390/molecules28124691)
Supplement: Supplementary file 1 [file molecules-28-04691-s001.zip › molecules-2395782-supplementary.pdf]

Supplementary Material Section S1:

To obtain the dataset for DeepBindGCN_RG_x, we excluded PDBbind v.2016 core set (CASF-2016 core set) and v.2013 core set from the PDBbind database and finally obtained 16,584 protein-ligand pairs. The dataset was divided into training and validation sets, containing 16,000 and 584 samples, respectively. The v.2013 core set (195 protein-ligand pairs) and PDBbind v.2016 core set (285 protein-ligand pairs) were kept as testing sets. The architecture is the same as the DeepBindGCN_RG; the entire training epoch is 2000; the models were saved every 100 epochs.

Supplementary Material Section S2:

Detailed procedure of MD, pocket MD, and metadynamics simulation

The initial protein-compound complexes were from the top score conformation Schrödinger docking. The ligand was edited by pymol software [1] to make it in the correct protonation state at pH 7.

We performed MD simulations for the TIPE3-compound complexes. We have cut the "FSSKSLALQAQKKILSKIAS" (amino acid 110 to 129) part of the TIPE3 to save the simulation resource. To save the computational resources, we have carried pocket MD for DNA-PKcs-compound and PD-L1-compound complexes by only keeping the binding pocket region for simulation. Metadynamics simulations can estimate binding free energy calculation to explore whether protein-ligand will bind in solution. Metadynamics relies on adding a bias potential to sample the free energy landscape along a specific collective variable of interest [2],[3]. Note that the binding free energy calculations from Metadynamics may only be suitable for detecting the general trend of binding in virtual screening.

The pocket MD is the same as the classical MD simulation, except that we only use the pocket region to reduce system size for simulation [4], which is inspired by a previous dynamic undocking (DUck) method [5]. An in-house script was used to extract the pocket region of the protein (here, we used 1.2nm within the binding ligand), and the N terminal and C terminal ends were capped with the ACE and NHE terminals, respectively. We applied position restraints to the ACE and NHE terminals to maintain the relative conformation of the pocket. MD or pocket MD simulation was carried out by Gromacs with AMBER-99SB force field [6,7]. The topology of the ligand and the partial charges of the ligand were generated by ACPYPE [8], which relies on Antechamber [9]. Firstly, we created a dodecahedron box and

put the target-ligand complex at the center. A minimum distance from the protein to the box edge was set to 1 nm. We filled the dodecahedron box with TIP3P water molecules [10], and the counter ions were added to neutralize the total charge using the Gromacs program tool [11]. The long-range electrostatic interactions under the periodic boundary conditions were calculated with the Particle Mesh Ewald approach [12]. A cutoff of 14 Å was used for van der Waals non-bonded interactions. Covalent bonds involving hydrogen atoms were constrained by applying the LINCS algorithm [13].

We performed the energy minimization steps with a step-size of 0.001ns, 100 ps simulation with an isothermal-isovolumetric ensemble (NVT), and 10ns simulation with the isothermal-isobaric ensemble (NPT) for water equilibrium. After that, a 40ns NPT production run (step size 2 fs) was carried out. The Parrinello-Rahman barostat and the modified Berendsen thermostat were used for simulation with a fixed temperature of 308 K and a pressure of 1 atm. RMSD and hydrogen bond number of the trajectory were calculated using Gromacs tools.

The simulation continued using the metadynamics approach to explore the free energy landscape. We carried 40ns metadynamics simulation with Plumed[14] patched Gromacs. The protein-ligand complex's interface coordination number of atoms was used as a collective variable (CV). The protein-ligand interface coordination numbers correlate with the numbers of atom contact, and a larger coordination number usually indicates that the protein-ligand is binding.

The coordination number *C* is defined as follows by Plumed:

$C = \sum_{i \in A} \sum_{j \in B} S_{ij}$ (1) and

$$S_{ij} = \frac{1 - \left(\frac{r_{ij} - d_0}{r_0}\right)^n}{1 - \left(\frac{r_{ij} - d_0}{r_0}\right)^m} \quad (2)$$

In the simulation, n was 6, m was 12, d_0 was 0 nm, and r_0 was 0.5 nm. d_0 is a parameter of the switching function. r_{ij} is the distance between atom i and atom j. The degrees of contact between two groups of atoms can be estimated by the above function(1) [14]. Metadynamics simulation for each protein-ligand system was performed for 40 ns. During the metadynamics simulation, Gaussian values were deposited every 1 ps with a height of 0.3 kJ/mol. The widths of the Gaussians were 5 for the coordination number. The free energy landscapes of the metadynamics simulations along the CV were generated by the Plumed program and plotted using Gnuplot [15].

Supplementary Figures:

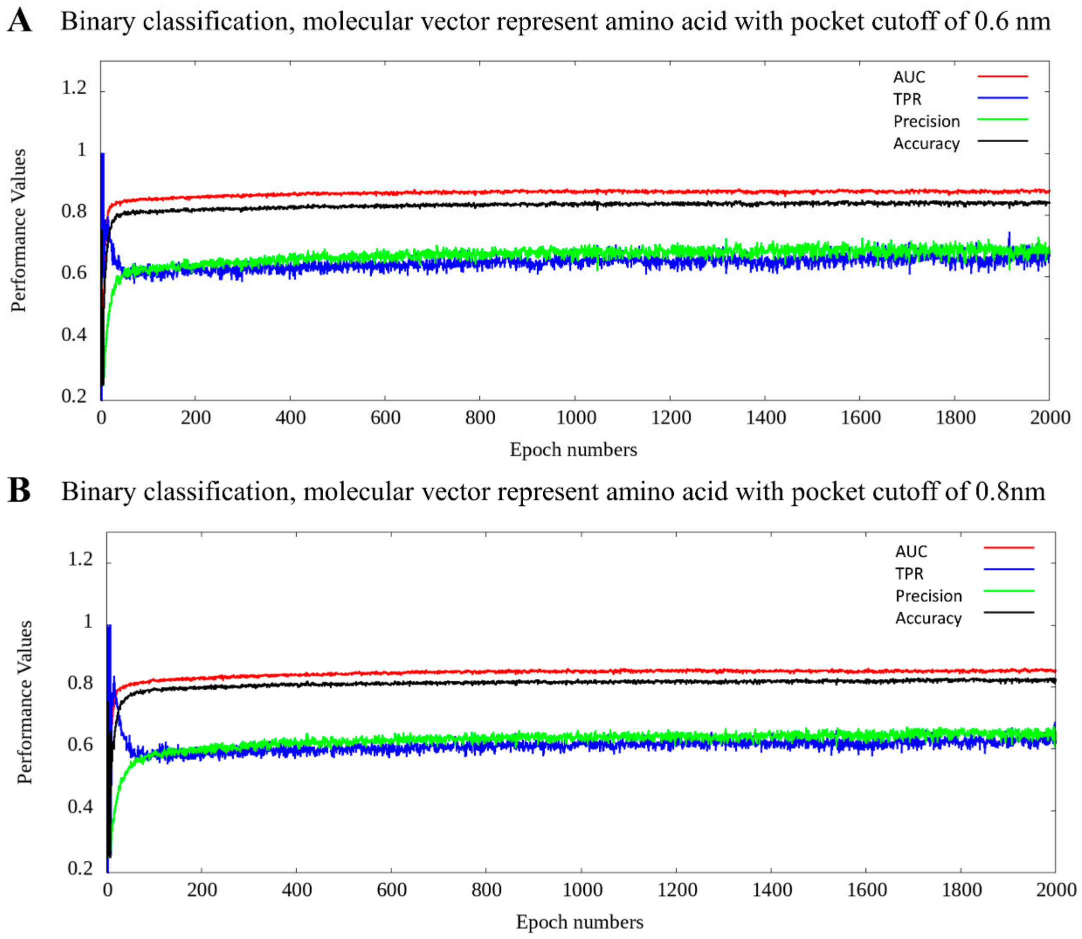

Figure S1. The DeepBindGCN_BC performance over the 2000 epoch training. A. pocket cutoff using 0.6 nm, B, pocket cutoff using 0.8 nm. The amino acid in the pocket is represented as a molecular vector.

A

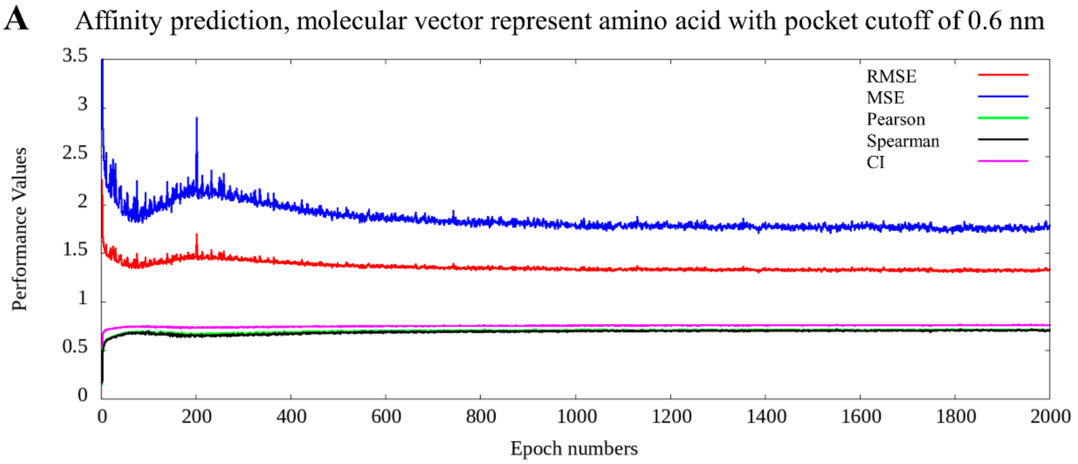

B

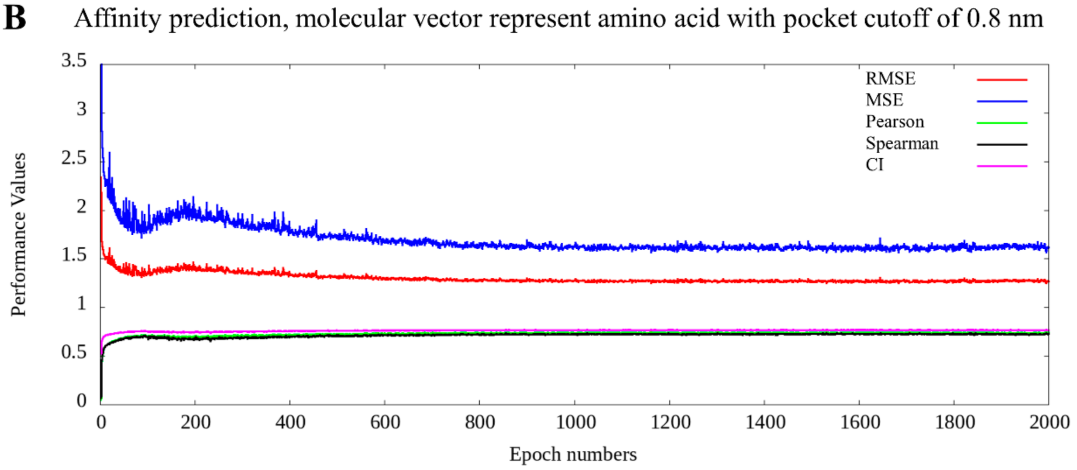

Figure S2. The DeepBindGCN_RG performance over the 2000 epoch training. A. pocket cutoff using 0.6 nm, B, pocket cutoff using 0.8 nm. The amino acids in the pocket are represented as molecular vectors.

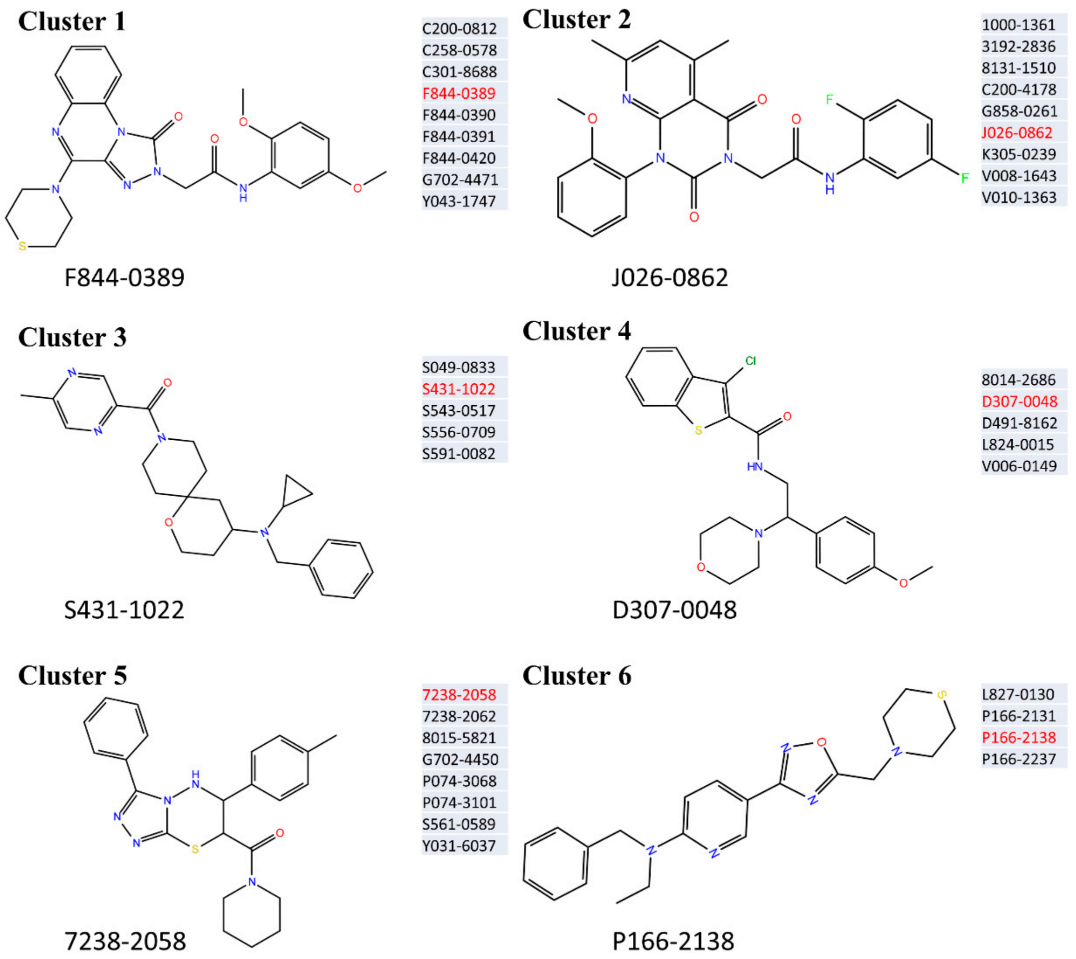

Figure S3. The six clusters and corresponding representative cluster center structure of top predicted candidates from DeepBindGCN_BC and DeepBindGCN_RG for the TIPE3.

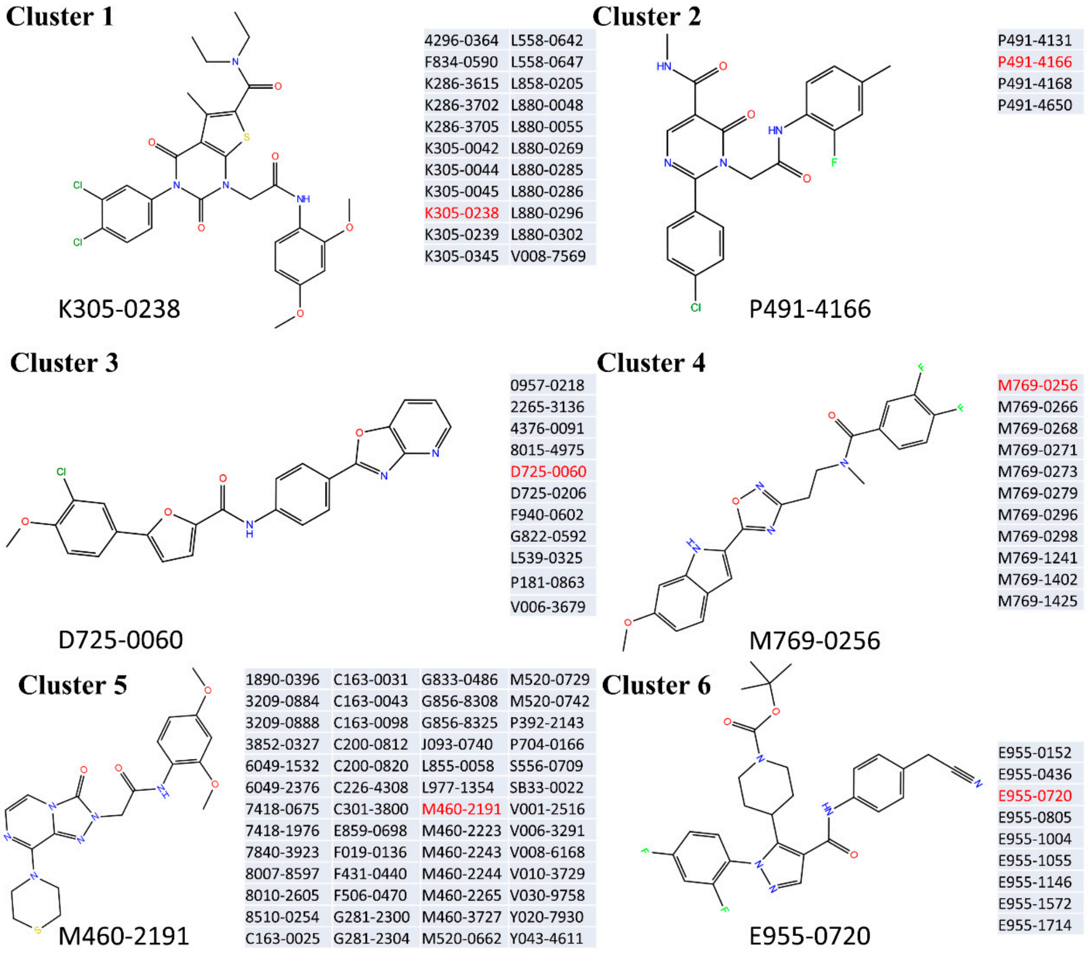

Figure S4. The six clusters and corresponding representative cluster center structure of top predicted candidates from DeepBindGCN_BC and DeepBindGCN_RG for the PD-L1.

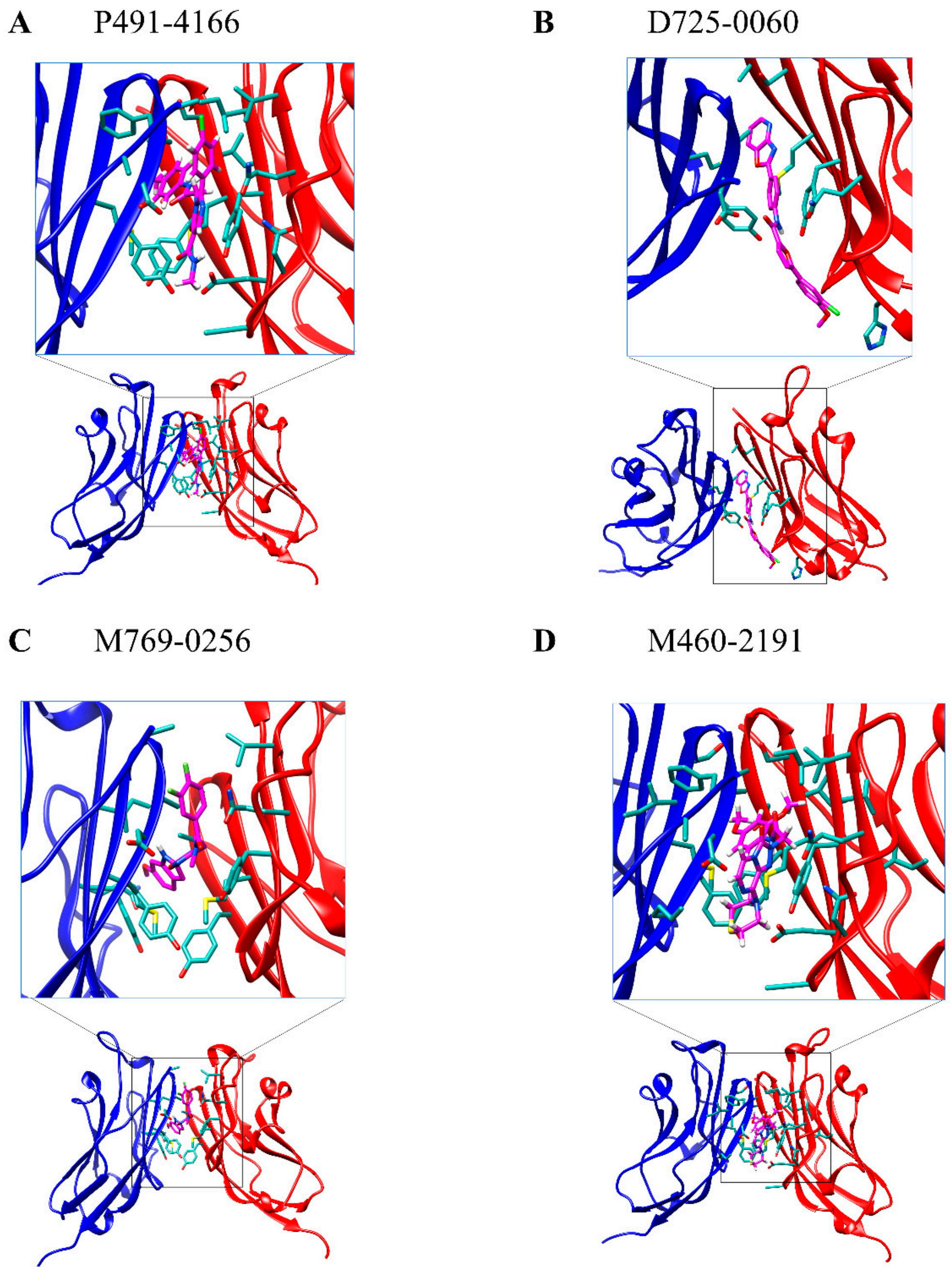

Figure S5. The snapshot and 2D plot of PD-L1 with representative cluster center compounds from docking.

A TIPE3

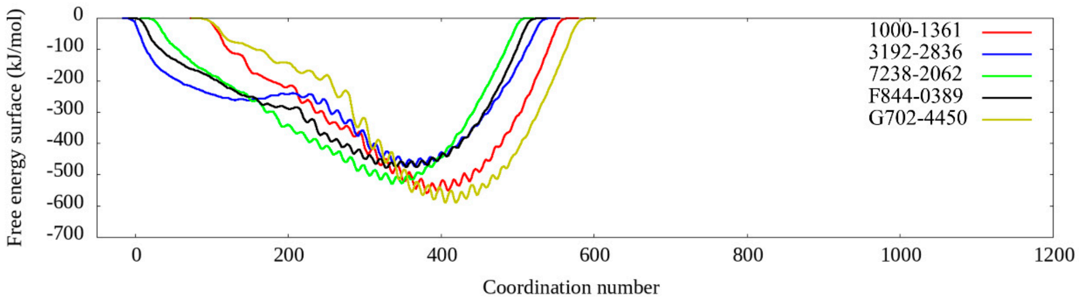

B PD-L1 dimer

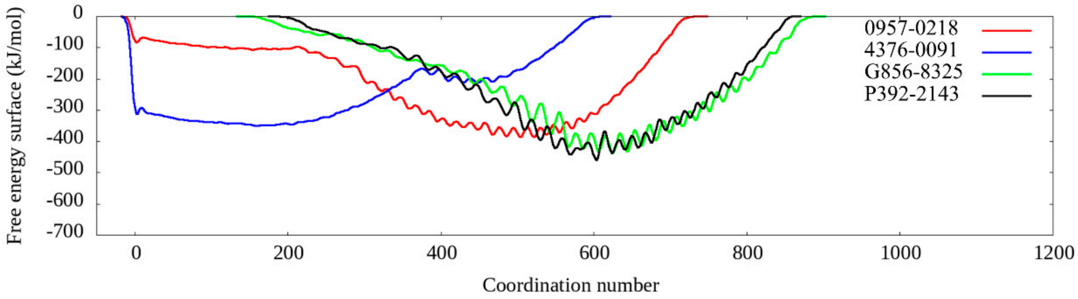

Figure S6. The calculated free energy landscape from metadynamics simulation for those candidates that have favorable binding with the given target. A, The calculated free energy landscape for TIPE3 with selected candidates; B, The calculated free energy landscape for PD-L1 with selected candidates.

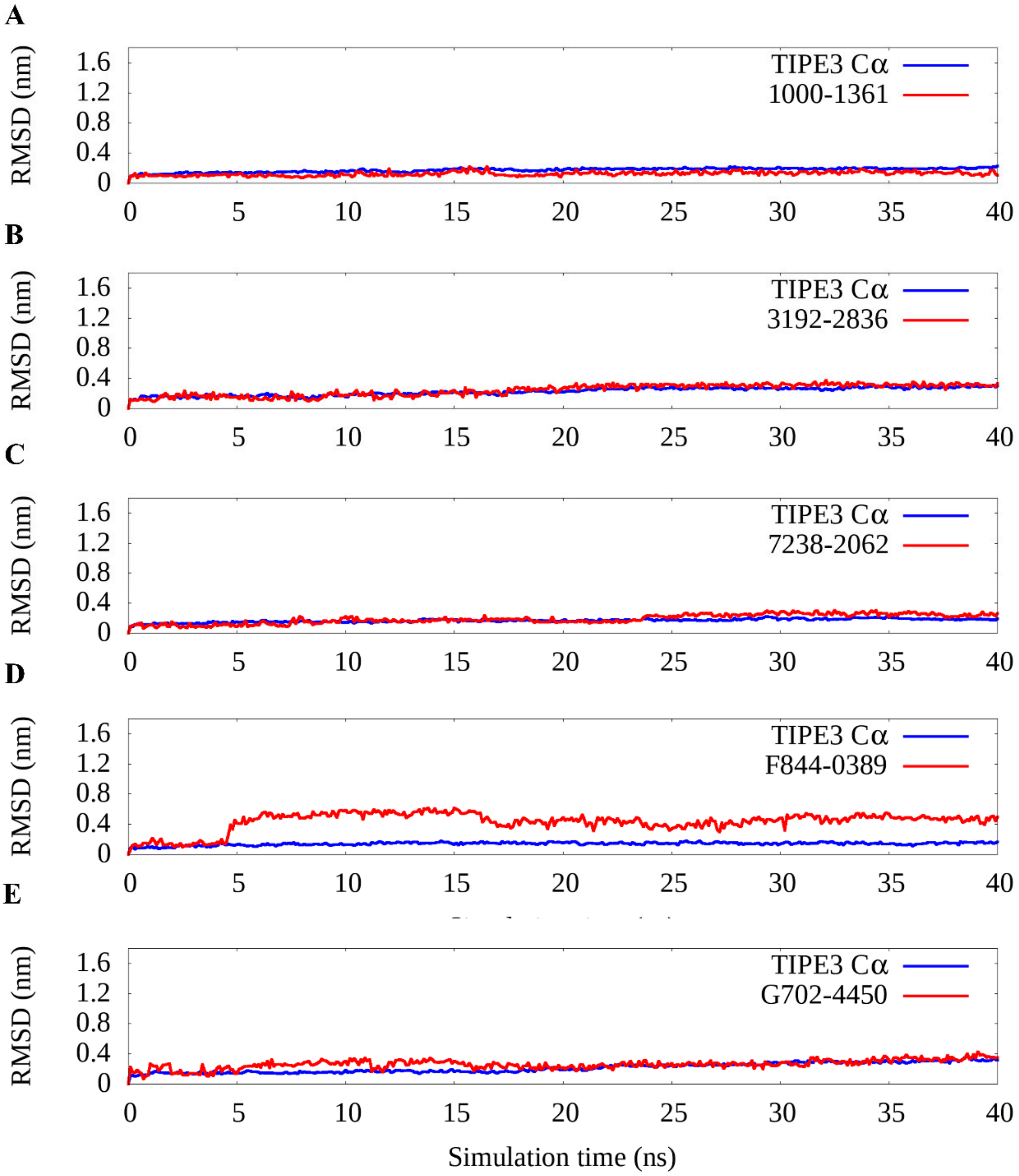

Figure S7. (A–E) The RMSD value of selected compounds with the TIPE3 during the MD simulation.

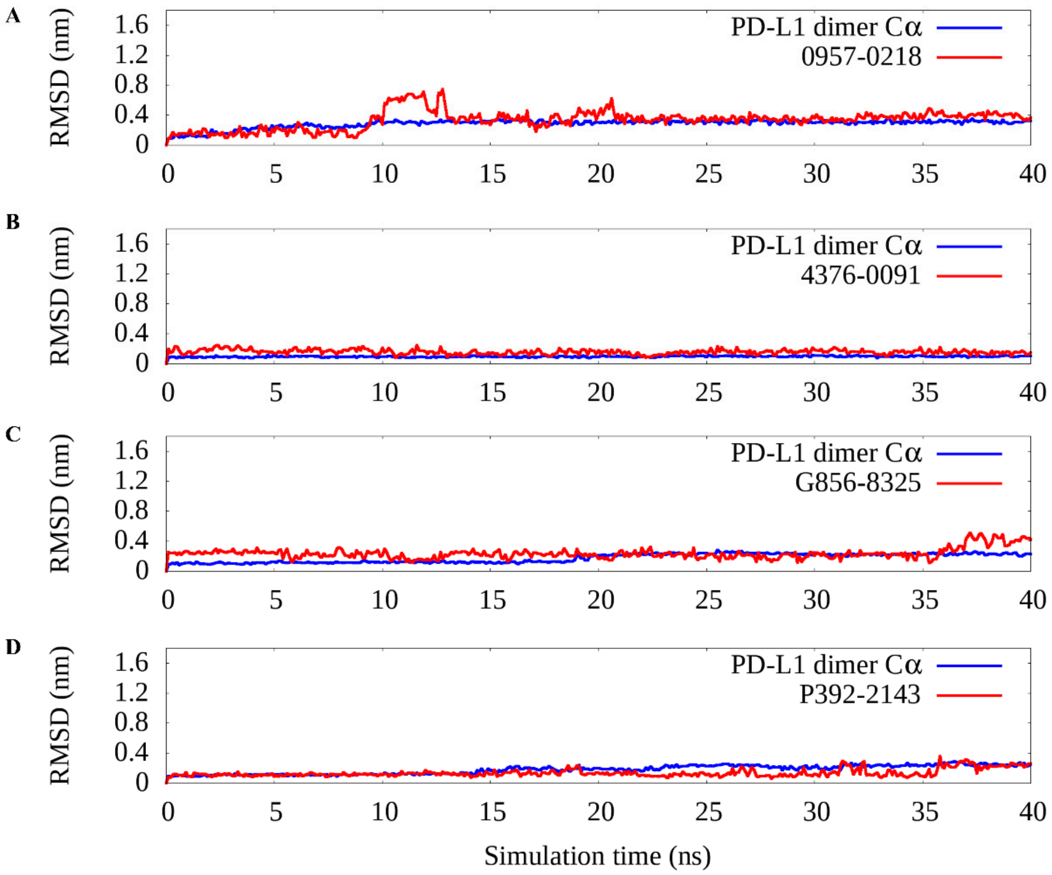

Figure S8. (A–D) The RMSD value of selected compounds with the PD-L1 during the MD simulation.

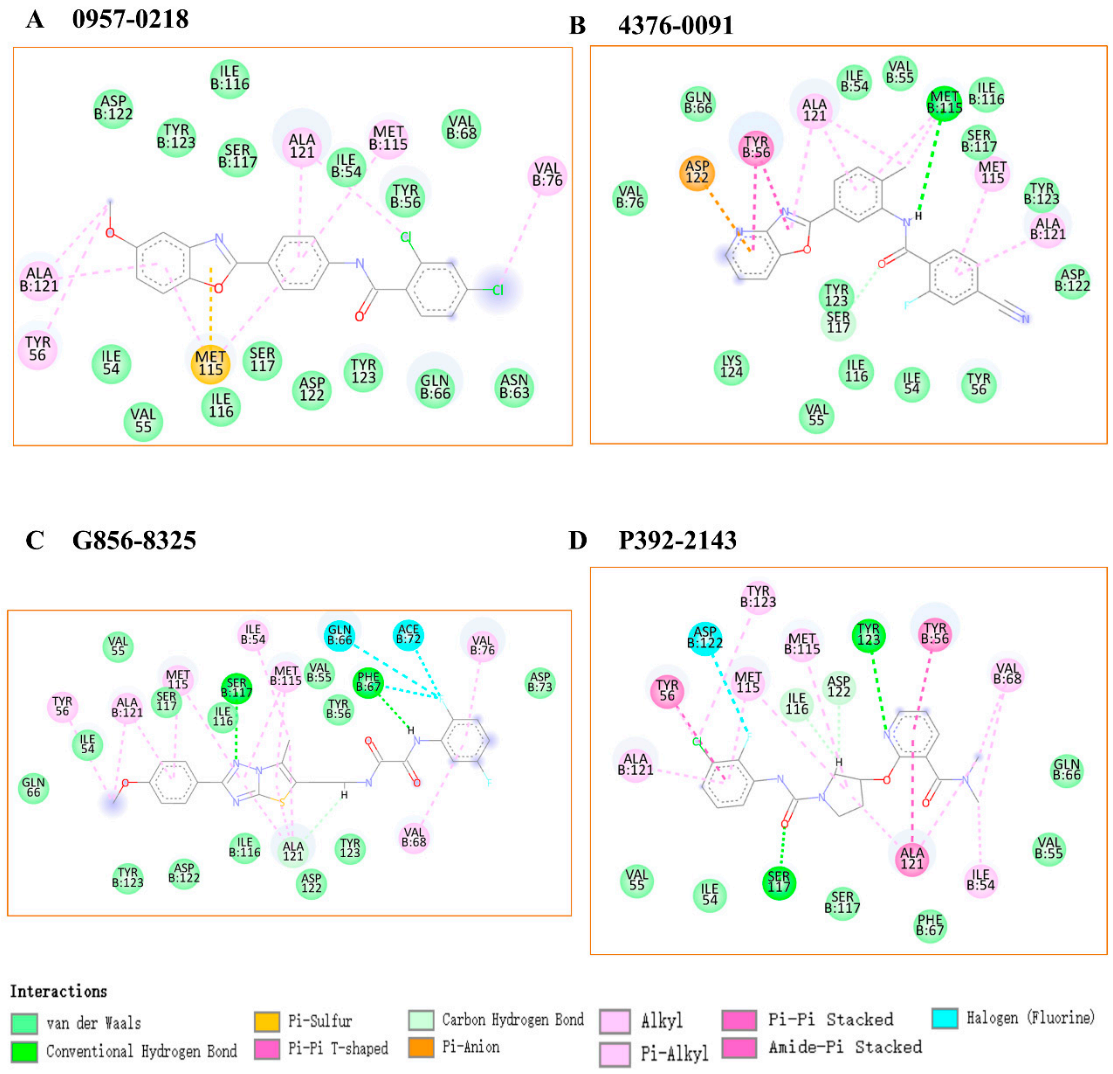

Figure S9. The 2D interaction snapshot of PD-L1 dimer with candidate compounds for the last frame from the MD simulation.

A Binary classification with one-hot like residue representation (pocket cutoff 0.6 nm)

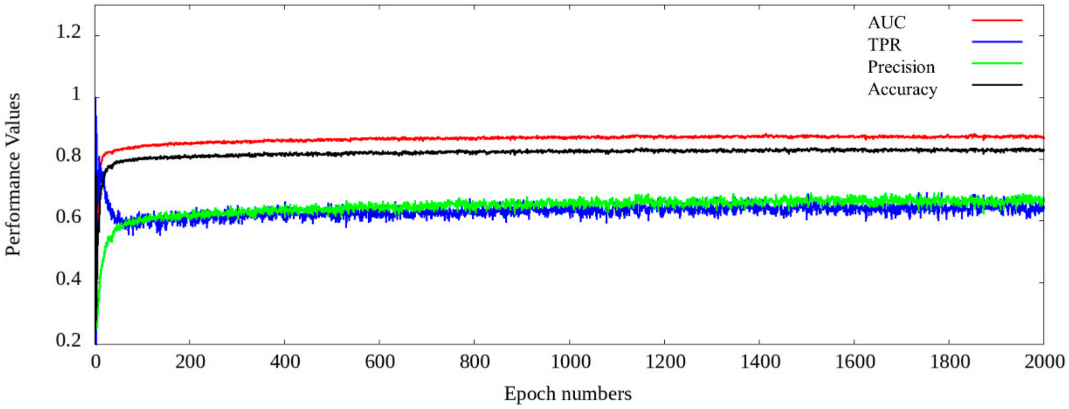

B Affinity prediction with one-hot like residue representation (pocket cutoff 0.8 nm)

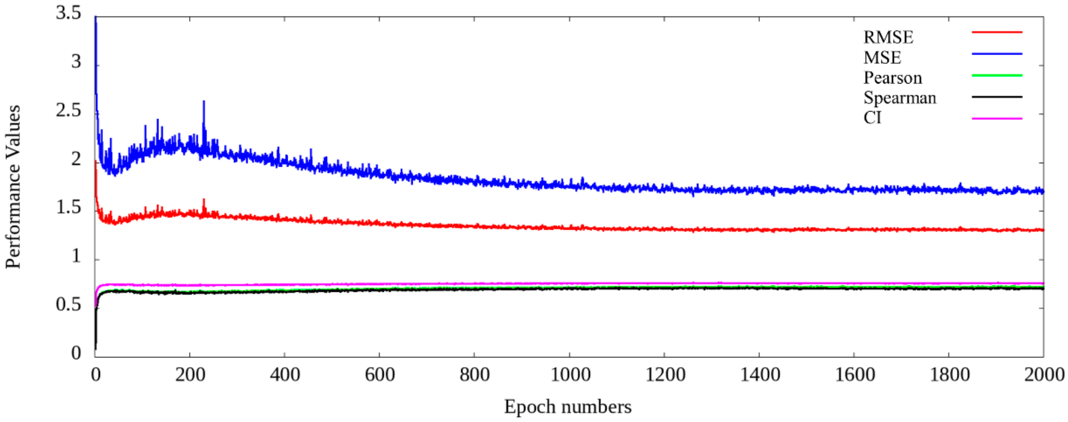

Figure S10. The performance of binary classification and affinity prediction models with onehot like residue representation over the 2000 epoch training. A. pocket cutoff using 0.6nm, B, pocket cutoff using 0.8nm. The amino acid in the pocket is represented as onehot representation.

Supplementary Tables:

Table S1. Performance of DeepBindGCN_BC on the test set during the training with epoch interval 100.

Pocket cutoff	Epoch	AUC	TPR	Precision	Accuracy
0.6nm	100	0.8493	0.6408	0.6096	0.8076
	200	0.8589	0.6138	0.6418	0.8178
	300	0.8626	0.6508	0.6246	0.8149
	400	0.8705	0.6254	0.6737	0.8306
	500	0.8676	0.6367	0.6524	0.8244
	600	0.8682	0.6350	0.6626	0.8279
	700	0.8750	0.6233	0.6831	0.8335
	800	0.8745	0.6508	0.6588	0.8284
	900	0.8789	0.6296	0.6837	0.8346
	1000	0.8767	0.6400	0.6802	0.8348
	1100	0.8703	0.6438	0.6747	0.8333
	1200	0.8744	0.6483	0.6801	0.8358
	1300	0.8719	0.6704	0.6632	0.8325
	1400	0.8755	0.6579	0.6765	0.8358
	1500	0.8790	0.6338	0.7071	0.8428
	1600	0.8752	0.6488	0.6929	0.8403
	1700	0.8775	0.6625	0.6780	0.8370
	1800	0.8738	0.6567	0.6915	0.8409
	1900	0.8778	0.6667	0.6917	0.8424
	2000	0.8788	0.6863	0.6767	0.8396
0.8nm	100	0.8222	0.5933	0.5901	0.7953
	200	0.8275	0.6021	0.5879	0.7950
	300	0.8367	0.5988	0.6186	0.8074
	400	0.8401	0.5875	0.6242	0.8084
	500	0.8403	0.6013	0.6304	0.8122
	600	0.8454	0.5904	0.6326	0.8119
	700	0.8443	0.6138	0.6236	0.8108
	800	0.8493	0.6154	0.6280	0.8127
	900	0.8513	0.6142	0.6420	0.8179
	1000	0.8493	0.6154	0.6422	0.8181
	1100	0.8502	0.6171	0.6329	0.8148
	1200	0.8559	0.6088	0.6543	0.8218
	1300	0.8560	0.6250	0.6394	0.8181
	1400	0.8492	0.6300	0.6340	0.8166
	1500	0.8539	0.6079	0.6479	0.8194
	1600	0.8550	0.6238	0.6531	0.8231
	1700	0.8517	0.6017	0.6600	0.8229
	1800	0.8489	0.6154	0.6527	0.8220
	1900	0.8546	0.6042	0.6648	0.8249

	2000	0.8537	0.6175	0.6552	0.8231

Table S2. Performance of DeepBindGCN_RG on test set during the training with epoch interval 100.

Pocket cutoff	Epoch	Rmse:	Mse	Pearson	Spearman	CI
0.6nm	100	1.3717	1.8816	0.7040	0.6968	0.7550
	200	1.4645	2.1449	0.6675	0.6524	0.7373
	300	1.4335	2.0550	0.6813	0.6684	0.7437
	400	1.4051	1.9744	0.6875	0.6725	0.7456
	500	1.3681	1.8716	0.6961	0.6860	0.7509
	600	1.3574	1.8426	0.7025	0.6915	0.7536
	700	1.3526	1.8296	0.7043	0.6927	0.7540
	800	1.3489	1.8195	0.7042	0.6949	0.7553
	900	1.3681	1.8717	0.7095	0.7003	0.7572
	1000	1.3308	1.7709	0.7108	0.7034	0.7591
	1100	1.3293	1.7670	0.7114	0.7038	0.7592
	1200	1.3417	1.8001	0.7084	0.7025	0.7585
	1300	1.3277	1.7628	0.7103	0.7025	0.7585
	1400	1.3483	1.8179	0.7067	0.6991	0.7573
	1500	1.3310	1.7715	0.7096	0.7030	0.7595
	1600	1.3291	1.7666	0.7118	0.7052	0.7599
	1700	1.3318	1.7738	0.7111	0.7043	0.7603
	1800	1.3327	1.7761	0.7106	0.7047	0.7602
	1900	1.3277	1.7627	0.7138	0.7067	0.7609
	2000	1.3361	1.7852	0.7141	0.7098	0.7628
0.8nm	100	1.3141	1.7270	0.7153	0.7137	0.7604
	200	1.3331	1.7772	0.7192	0.7062	0.7572
	300	1.2859	1.6534	0.7392	0.7256	0.7677
	400	1.2966	1.6810	0.7276	0.7122	0.7620
	500	1.2672	1.6059	0.7413	0.7311	0.7702
	600	1.2465	1.5538	0.7497	0.7396	0.7752
	700	1.2558	1.5770	0.7423	0.7309	0.7714
	800	1.2201	1.4885	0.7580	0.7463	0.7779
	900	1.2135	1.4725	0.7560	0.7498	0.7786
	1000	1.2039	1.4494	0.7580	0.7500	0.7796
	1100	1.2298	1.5124	0.7467	0.7393	0.7737
	1200	1.1914	1.4195	0.7617	0.7475	0.7789
	1300	1.1991	1.4378	0.7574	0.7448	0.7775
	1400	1.2004	1.4410	0.7570	0.7446	0.7778
	1500	1.2039	1.4494	0.7563	0.7435	0.7770
	1600	1.2184	1.4844	0.7484	0.7370	0.7726
	1700	1.2162	1.4791	0.7497	0.7400	0.7752
	1800	1.2176	1.4825	0.7501	0.7372	0.7741
	1900	1.2083	1.4599	0.7518	0.7398	0.7745
	2000	1.2107	1.4657	0.7518	0.7410	0.7756

Table S3. The performance of DeepBindGCN_BC (pocket cutoff 0.6 nm) on the DUD.E dataset.

PDBID	AUC	TPR	Precision	Accuracy	MCC	data_size	pos_size	neg_size
3BWM	1.0000	0.8537	1.0000	0.8571	0.3492	42	41	1
1ZW5	0.5765	0.0118	1.0000	0.2500	0.0535	112	85	27
2AA2	0.7052	0.2217	1.0000	0.2290	0.0515	214	212	2
3KRJ	0.9378	0.7558	1.0000	0.7589	0.1944	394	389	5
3L3M	0.8029	0.5301	1.0000	0.5355	0.1125	1057	1045	12
2OWB	0.4205	0.0044	1.0000	0.1722	0.0273	273	227	46
3KBA	0.1230	0.3615	0.9975	0.3611	-0.0396	1127	1126	1
3CCW	0.7652	0.5878	0.9969	0.5920	0.1124	549	541	8
3PBL	0.6470	0.8780	0.9954	0.8748	0.0565	2228	2214	14
3BQD	0.5618	0.7802	0.9949	0.7776	0.0212	998	992	6
3G0E	0.8057	0.6887	0.9924	0.6899	0.1338	387	379	8
830C	0.6763	0.7968	0.9902	0.7922	0.0906	1670	1644	26
2CNK	0.7350	0.1928	0.9891	0.2495	0.1118	509	472	37
1XL2	0.8517	0.4639	0.9887	0.4910	0.1817	1607	1511	96
3EQH	0.6921	0.2403	0.9867	0.2656	0.0704	320	308	12
2ZEC	0.6770	0.3122	0.9857	0.3544	0.1373	237	221	16
2AM9	0.5055	0.8199	0.9835	0.8094	0.0103	1107	1088	19
1BCD	0.4933	0.1675	0.9822	0.1753	-0.0191	2002	1976	26
3D0E	0.8424	0.6498	0.9809	0.6692	0.3015	260	237	23
2OI0	0.5505	0.5676	0.9808	0.5665	0.0250	1384	1353	31
1MV9	0.6055	0.8322	0.9806	0.8199	0.0465	311	304	7
3LPB	0.4851	0.3690	0.9789	0.3760	0.0112	258	252	6
2QD9	0.6189	0.8174	0.9758	0.8036	0.0902	2291	2218	73
3HMM	0.5769	0.7489	0.9670	0.7314	-0.0420	242	235	7
2H7L	0.5769	0.7489	0.9670	0.7314	-0.0420	242	235	7
3L5D	0.8266	0.9133	0.9665	0.8892	0.3445	641	600	41
3EML	0.6269	0.4002	0.9665	0.4221	0.0847	3288	3096	192
2FSZ	0.8597	0.9173	0.9661	0.8948	0.4686	1492	1366	126
2AYW	0.7089	0.2182	0.9638	0.2946	0.1154	1093	976	117
3FRJ	0.4572	0.1207	0.9633	0.1446	-0.0093	899	870	29
2GTK	0.3925	0.7785	0.9626	0.7564	-0.0550	1334	1291	43
3LQ8	0.5867	0.6042	0.9621	0.6006	0.0583	353	336	17
1SJ0	0.8025	0.7057	0.9617	0.7078	0.2678	1451	1315	136
3BGS	0.5118	0.8109	0.9602	0.7863	0.0055	248	238	10
3CJO	0.6768	0.5109	0.9592	0.5377	0.1784	305	276	29
3CHP	0.6478	0.8295	0.9567	0.8038	0.1265	367	346	21
2P2I	0.6366	0.6983	0.9541	0.6840	0.0751	2462	2320	142
1UDT	0.7414	0.7536	0.9531	0.7413	0.2310	1063	970	93
3D4Q	0.7178	0.8202	0.9524	0.7971	0.2392	345	317	28
3KL6	0.5061	0.4785	0.9492	0.4817	0.0082	3340	3164	176

2ETR	0.5402	0.6804	0.9490	0.6667	0.0766	234	219	15
1YPE	0.6432	0.4269	0.9485	0.4636	0.1341	2541	2286	255
3HL5	0.5043	0.7300	0.9481	0.7103	0.0873	107	100	7
3BKL	0.6001	0.6494	0.9479	0.6382	0.0621	868	813	55
3BIZ	0.5069	0.9005	0.9476	0.8602	0.1302	236	221	15
2P54	0.6506	0.8819	0.9469	0.8441	0.1672	1174	1092	82
1SQT	0.4882	0.1387	0.9455	0.2220	0.0640	419	375	44
2VT4	0.5656	0.4779	0.9429	0.5014	0.1192	726	657	69
2HZI	0.8138	0.6895	0.9400	0.7059	0.3660	493	409	84
3CQW	0.6763	0.6037	0.9367	0.5991	0.0845	641	588	53
1B9V	0.5898	0.5463	0.9333	0.5531	0.0962	226	205	21
2I78	0.5760	0.8865	0.9330	0.8364	0.0924	2194	2027	167
2RGP	0.8190	0.7463	0.9322	0.7538	0.4423	2027	1620	407
1H00	0.6143	0.6033	0.9302	0.5992	0.0957	1462	1326	136
3G6Z	0.6409	0.8668	0.9299	0.8221	0.2480	444	398	46
3F07	0.7875	0.8307	0.9298	0.8112	0.4865	392	319	73
2OF2	0.6871	0.5484	0.9265	0.5736	0.1923	1067	919	148
3E37	0.5940	0.5236	0.9249	0.5242	0.0298	1591	1459	132
1LI4	0.5538	0.6769	0.9167	0.6429	-0.0683	70	65	5
2ICA	0.6365	0.8025	0.9155	0.7507	-0.0174	353	324	29
1C8K	0.5284	0.2530	0.9130	0.2889	-0.0201	90	83	7
3NXO	0.4664	0.7588	0.8970	0.7023	-0.0601	1253	1136	117
2ZNP	0.5381	0.8696	0.8960	0.7917	-0.0377	792	713	79
3MAX	0.6439	0.8160	0.8915	0.7537	0.1293	475	413	62
2HV5	0.4378	0.2261	0.8896	0.2895	0.0062	684	606	78
1LRU	0.2677	0.0925	0.8889	0.1215	-0.1082	181	173	8
3BZ3	0.2466	0.8713	0.8889	0.7857	-0.1196	112	101	11
2OJ9	0.5570	0.4316	0.8703	0.4732	0.0846	448	373	75
3EL8	0.6774	0.7549	0.8689	0.7071	0.2130	1560	1273	287
2ZDT	0.4340	0.5495	0.8605	0.5156	-0.1428	225	202	23
1E66	0.6548	0.5015	0.8551	0.5504	0.1826	2122	1635	487
1NJS	0.5897	0.9808	0.8500	0.8438	0.3721	64	52	12
3LAN	0.5831	0.6461	0.8490	0.6141	0.0877	1459	1201	258
1Q4X	0.1732	0.5610	0.8415	0.5127	-0.2101	275	246	29
3ODU	0.7184	0.8372	0.8372	0.7544	0.3372	57	43	14
3NY8	0.4513	0.5622	0.8325	0.5373	-0.0230	737	619	118
2NNQ	0.8364	0.9362	0.8000	0.7778	0.3251	63	47	16
3M2W	0.6844	0.6848	0.7826	0.6491	0.2384	265	184	81
1S3B	0.4886	0.8377	0.7812	0.6906	0.0092	585	456	129
2I0E	0.6469	0.6141	0.7793	0.6046	0.1795	521	368	153
2OJG	0.5857	0.8148	0.7765	0.7069	0.2821	116	81	35
1SYN	0.3875	0.2786	0.7724	0.3054	-0.1812	465	402	63
1J4H	0.5961	0.7818	0.7500	0.6609	0.1546	233	165	68
1UYG	0.3655	0.6705	0.7375	0.5575	-0.1548	113	88	25

2E1W	0.5014	0.7404	0.7333	0.6233	0.0743	146	104	42
3C4F	0.5703	0.6789	0.7115	0.5877	0.0609	473	327	146
3LN1	0.3907	0.2277	0.7099	0.3114	-0.1247	2174	1730	444
1D3G	0.4088	0.2247	0.7083	0.3746	-0.0149	315	227	88
1KVO	0.2119	0.0909	0.6957	0.1211	-0.3277	190	176	14
1W7X	0.2159	0.1508	0.6866	0.1860	-0.3103	344	305	39
3KGC	0.3955	0.5123	0.6631	0.4702	-0.1092	689	488	201
2V3F	0.3983	0.4000	0.6471	0.4512	-0.0424	82	55	27
2OYU	0.5191	0.0976	0.6023	0.6745	0.1350	1613	543	1070
2AZR	0.4730	0.0458	0.4815	0.3478	-0.0906	437	284	153
3NF7	0.5717	0.6973	0.4388	0.5121	0.0841	453	185	268
1L2S	0.6311	0.5510	0.4355	0.5714	0.1299	133	49	84
3NXU	0.3981	0.3135	0.4241	0.4088	-0.1733	570	303	267
1QW6	0.4563	0.0155	0.3846	0.1772	-0.2046	395	322	73
1R9O	0.3632	0.1172	0.3617	0.5078	-0.0749	321	145	176
1VSO	0.3137	0.4706	0.2712	0.3423	-0.2617	371	136	235
2B8T	0.4948	0.0000	nan	0.5397	0.0000	126	58	68
3F9M	0.5357	0.0000	nan	0.0886	0.0000	158	144	14

Table S4. The performance of DeepBindGCN_RG (pocket cutoff 0.8 nm) on the DUD.E dataset.

PDBID	Rmse	Mse	Pearson	Spearman	CI	data_size
3BIZ	0.6866	0.4714	0.1794	0.1800	0.5570	221
2AZR	0.7134	0.5089	0.2293	0.2654	0.5903	284
1UYG	0.7880	0.6209	0.3155	0.2981	0.6089	88
3M2W	0.7958	0.6334	0.3754	0.3063	0.6073	184
3EQH	0.8114	0.6584	0.3547	0.3277	0.6159	308
2ETR	0.8119	0.6592	0.2780	0.2687	0.5961	219
3F9M	0.8177	0.6686	0.1705	0.1740	0.5611	144
1KVO	0.8184	0.6697	0.1789	0.1481	0.5510	176
1SQT	0.8194	0.6715	0.2473	0.2282	0.5777	375
3D0E	0.8439	0.7122	0.2704	0.2272	0.5797	237
3L5D	0.8480	0.7191	0.3180	0.3432	0.6187	600
1LRU	0.8956	0.8021	0.2213	0.2362	0.5805	173
3NF7	0.9010	0.8119	0.1790	0.1021	0.5353	185
3HMM	0.9035	0.8163	0.0380	0.0055	0.5010	235
2ICA	0.9056	0.8201	0.3269	0.3630	0.6210	324
2HZI	0.9088	0.8258	0.5412	0.5701	0.6958	409
3KGC	0.9121	0.8319	-0.0222	0.0049	0.5013	488
2HV5	0.9258	0.8572	0.0512	0.0530	0.5178	606
3EL8	0.9303	0.8654	0.2629	0.2570	0.5875	1271
2OJG	0.9386	0.8810	0.5505	0.5713	0.7045	81
1D3G	0.9397	0.8831	0.0503	0.0742	0.5269	227
1BCD	0.9496	0.9017	0.3138	0.2846	0.5974	1976
2V3F	0.9621	0.9256	0.3420	0.2885	0.5987	55
3CCW	0.9665	0.9341	0.2556	0.2955	0.6004	541
2QD9	0.9730	0.9468	0.3492	0.3509	0.6196	2218
3KRJ	0.9770	0.9545	0.2654	0.2395	0.5826	389
3CQW	0.9779	0.9562	0.2804	0.2742	0.5933	588
2ZNP	0.9779	0.9564	0.1656	0.1517	0.5510	713
2OF2	0.9816	0.9635	0.2678	0.2355	0.5797	919
830C	0.9833	0.9668	0.2000	0.1883	0.5641	1644
3LAN	0.9854	0.9709	0.1809	0.1732	0.5596	1201
2OJ9	0.9918	0.9836	0.4426	0.4041	0.6388	373
3MAX	0.9936	0.9873	0.0286	0.0379	0.5130	413
1J4H	0.9965	0.9930	-0.1850	-0.1821	0.4383	165
3G0E	0.9967	0.9935	0.0037	-0.0001	0.4966	379
1UDT	0.9988	0.9976	0.4255	0.4115	0.6419	970
3FRJ	1.0053	1.0107	0.3219	0.3558	0.6219	870
3LN1	1.0114	1.0229	0.1226	0.1406	0.5467	1724
2OYU	1.0176	1.0356	0.0176	-0.0037	0.4991	542
1MV9	1.0219	1.0443	-0.0451	-0.0704	0.4770	302
2I0E	1.0248	1.0503	0.1046	0.0637	0.5211	368

3G6Z	1.0335	1.0681	0.3708	0.3808	0.6315	398
2P54	1.0358	1.0729	-0.0942	-0.0807	0.4732	1092
3C4F	1.0446	1.0912	0.2187	0.2043	0.5695	327
2OI0	1.0482	1.0987	0.2427	0.2715	0.5921	1353
3BZ3	1.0552	1.1134	0.4161	0.2853	0.5996	101
2FSZ	1.0561	1.1154	0.1761	0.1903	0.5635	1366
3L3M	1.0662	1.1368	0.2866	0.2893	0.5993	1045
2I78	1.0709	1.1468	0.2367	0.2588	0.5878	2027
2NNQ	1.0723	1.1498	-0.0430	0.0930	0.5363	47
1W7X	1.0802	1.1669	-0.1663	-0.1278	0.4586	305
1H00	1.0811	1.1687	0.1471	0.1481	0.5504	1326
3CJO	1.1029	1.2165	0.0636	0.0040	0.5023	276
2GTK	1.1122	1.2370	0.1875	0.1640	0.5554	1291
3E37	1.1124	1.2374	0.3073	0.2869	0.5971	1458
3LQ8	1.1194	1.2531	0.2573	0.2449	0.5826	336
2AA2	1.1231	1.2614	0.1248	0.1253	0.5415	212
3BQD	1.1239	1.2632	0.2318	0.2555	0.5849	992
2P2I	1.1261	1.2682	0.1844	0.1634	0.5554	2320
3KL6	1.1401	1.2998	0.3700	0.3639	0.6240	3164
3D4Q	1.1457	1.3127	0.3178	0.3248	0.6096	317
3NXU	1.1501	1.3226	0.1605	0.0808	0.5280	301
1LI4	1.1507	1.3240	0.1877	0.1849	0.5623	65
3KBA	1.1584	1.3418	0.2295	0.1383	0.5450	1126
3BKL	1.1908	1.4180	0.1231	0.1792	0.5606	813
3CHP	1.2059	1.4543	0.2302	0.2447	0.5830	346
3F07	1.2073	1.4576	0.0710	0.0406	0.5144	319
2ZEC	1.2095	1.4629	0.1899	0.2193	0.5701	221
2OWB	1.2178	1.4830	-0.0745	0.0285	0.5094	227
1SYN	1.2312	1.5158	0.1394	0.1719	0.5579	402
2ZDT	1.2347	1.5244	0.0851	0.0698	0.5228	202
3PBL	1.2370	1.5302	0.2382	0.2288	0.5772	2212
3NY8	1.2412	1.5405	0.0940	0.0653	0.5224	612
2CNK	1.2492	1.5606	0.1769	0.2146	0.5712	472
1VSO	1.2498	1.5619	-0.0160	-0.0015	0.5018	136
1R9O	1.2565	1.5787	0.0482	-0.0124	0.4960	143
2AYW	1.2632	1.5957	0.2006	0.2593	0.5870	975
1E66	1.2722	1.6184	0.0327	0.0638	0.5212	1635
3LPB	1.2798	1.6379	0.2051	0.2103	0.5699	252
1XL2	1.2904	1.6652	0.2034	0.2053	0.5695	1510
2RGP	1.3472	1.8149	-0.0431	-0.0291	0.4903	1620
1C8K	1.3568	1.8408	-0.2455	-0.1909	0.4346	83
2AM9	1.3654	1.8643	0.0073	0.0170	0.5063	1086
1SJ0	1.3782	1.8994	0.2670	0.2893	0.5966	1315
1ZW5	1.3800	1.9045	-0.0774	-0.1686	0.4433	85

1L2S	1.4351	2.0594	0.3744	0.2842	0.6015	49
1Q4X	1.5252	2.3263	0.1688	0.1581	0.5511	246
3EML	1.5608	2.4360	0.2073	0.1968	0.5664	3094
2E1W	1.5950	2.5441	0.0564	0.3352	0.6280	104
3BWM	1.6102	2.5927	0.5564	0.5051	0.6847	41
2VT4	1.6758	2.8084	-0.0898	-0.1105	0.4632	656
3NXO	1.7128	2.9336	0.1981	0.2121	0.5701	1136
3BGS	1.7390	3.0243	0.2201	0.2084	0.5703	238
1B9V	1.7737	3.1460	-0.0314	-0.0547	0.4799	205
3ODU	1.8775	3.5251	-0.5341	-0.5619	0.3000	43
1YPE	1.9325	3.7347	0.1137	0.0743	0.5261	2286
2H7L	1.9446	3.7814	-0.0870	-0.1644	0.4295	44
2B8T	2.0177	4.0713	0.4273	0.3408	0.6174	58
1QW6	2.1217	4.5014	0.0362	0.0522	0.5173	322
1NJS	2.2257	4.9538	-0.0284	-0.0702	0.4784	52
3HL5	2.3382	5.4670	0.1609	0.2207	0.5754	100
1S3B	2.6465	7.0038	-0.1133	-0.0348	0.4883	455
Average	1.1893	1.5440	0.1655	0.1626	0.5557	668.8725

Table S5. The top predicted candidates from DeepBindGCN_BC and DeepBindGCN_RG for the PD-L1 dimer.

Compound ID	DeepBindGCN_BC	DeepBindGCN_RG	Schrödinger score
M769-0298	1.0000	8.6240	-7.0316
8510-0254	1.0000	8.6550	-8.5080
7840-3923	1.0000	8.6628	-8.9960
K305-0238	1.0000	8.7054	None
M769-0279	1.0000	8.7637	-8.0958
E955-1714	1.0000	8.8704	None
K286-3615	1.0000	8.6922	-6.2060
P181-0863	1.0000	8.6661	-7.0520
E955-1146	1.0000	8.6790	None
4376-0091	1.0000	8.6287	-9.5048
P491-4168	1.0000	8.7444	-4.4769
P491-4131	1.0000	8.7610	-7.1448
P491-4650	1.0000	8.6005	-6.7671
4296-0364	1.0000	8.6700	None
L880-0302	1.0000	8.6232	-5.7479
C163-0031	1.0000	9.0551	-7.3762
F019-0136	1.0000	8.9002	-5.3502
E955-1004	1.0000	9.1059	None
K305-0239	1.0000	8.6878	None
E955-1572	1.0000	8.7449	None
V010-3729	1.0000	8.6142	-9.5620
C301-3800	1.0000	8.6228	None
G833-0486	0.9999	8.6179	-8.4276
7418-0675	0.9999	8.6696	-8.5439
M769-0266	0.9999	8.9623	-8.9308
0957-0218	0.9999	8.6395	-9.1960
M769-1241	0.9999	8.7414	-9.2589
K286-3705	0.9999	8.6012	-5.9413
L880-0055	0.9999	8.6495	-7.0515
E955-0152	0.9999	8.7428	None
1890-0396	0.9999	8.6412	-7.6736
E955-0720	0.9999	8.6566	None
G281-2300	0.9999	8.6932	-2.7696
K305-0345	0.9999	8.6874	None
F506-0470	0.9999	8.6808	None
P491-4166	0.9999	8.6334	-6.9812
6049-2376	0.9999	8.9938	-6.9023
M769-0273	0.9999	8.6812	-8.5714
L880-0048	0.9999	8.6338	-5.7588
M769-0271	0.9999	8.8637	-6.3845

3209-0888	0.9998	8.6119	-6.8953
M769-0296	0.9998	8.6302	-7.7200
M769-0268	0.9998	8.8134	-4.7701
M769-0256	0.9998	8.7986	-8.5208
F940-0602	0.9997	8.6125	-8.9528
D725-0206	0.9997	8.6493	-8.5324
G822-0592	0.9997	8.6111	-5.7201
C163-0098	0.9996	8.7322	-8.3344
6049-1532	0.9996	8.7974	-6.2506
K305-0045	0.9996	8.6580	None
C200-0820	0.9996	8.7744	None
L558-0642	0.9995	8.6285	None
E859-0698	0.9995	8.6636	-9.5635
K286-3702	0.9995	8.6177	None
E955-0436	0.9995	8.7842	None
K305-0044	0.9994	8.7119	None
3852-0327	0.9994	8.6303	-8.1867
7418-1976	0.9994	8.6795	-9.0079
L558-0647	0.9994	8.7484	None
M460-2243	0.9993	8.6750	-7.3363
F834-0590	0.9993	8.6778	-8.7997
V001-2516	0.9992	8.6688	-9.6745
L880-0269	0.9992	8.6402	-8.2118
L880-0296	0.9992	8.7370	-4.7731
Y043-4611	0.9992	8.6051	-7.6287
V006-3291	0.9992	8.7025	-9.4725
M460-2244	0.9991	8.9798	-7.8858
3209-0884	0.9990	8.6122	None
L855-0058	0.9989	8.6996	-4.5421
M769-1425	0.9989	8.7582	-7.3411
C200-0812	0.9987	8.9092	-6.5423
G281-2304	0.9986	8.6122	-1.3766
8010-2605	0.9985	8.6211	None
G856-8325	0.9984	8.7683	-9.6361
C163-0025	0.9981	8.8068	-8.0547
V030-9758	0.9981	8.9498	-8.9287
V006-3679	0.9979	8.7418	-9.2080
M460-2265	0.9978	8.6296	-8.4071
G856-8308	0.9977	8.6416	-9.0176
L880-0285	0.9976	8.7407	-5.1432
P704-0166	0.9974	8.6151	-7.4346
L539-0325	0.9973	8.6117	-7.8659
L977-1354	0.9973	8.6063	-2.7656
8007-8597	0.9972	8.6505	-7.5859

2265-3136	0.9972	8.6034	None
M520-0742	0.9970	8.6904	-8.8531
V008-6168	0.9968	8.8348	-8.5468
M520-0662	0.9967	8.6738	-8.7562
8015-4975	0.9964	8.7747	-5.5618
C226-4308	0.9960	8.8256	-7.5766
E955-0805	0.9949	8.6627	None
L880-0286	0.9948	8.6384	-5.7523
F431-0440	0.9943	8.6648	-7.3802
Y020-7930	0.9941	8.6259	-5.6173
M460-3727	0.9939	8.6979	-8.5876
P392-2143	0.9933	8.7914	-9.0883
L858-0205	0.9932	8.6213	-3.2103
M460-2223	0.9926	8.6092	-6.9175
M460-2191	0.9923	8.6177	-6.6068
J093-0740	0.9922	8.6521	-8.4309
M769-1402	0.9922	8.6924	-6.9686
SB33-0022	0.9917	8.6459	-6.0260
S556-0709	0.9917	8.7039	-7.0693
M520-0729	0.9914	8.8291	-9.1131
C163-0043	0.9909	8.6221	-8.2081
K305-0042	0.9908	8.8660	-6.9674
V008-7569	0.9902	8.7126	-5.2661
E955-1055	0.9901	8.6774	None
D725-0060	0.9900	8.6192	-6.8281

Table S6. The performance of DeepBindGCN_RG_x with different training epoch on the PDBbind v.2016 core set (CASF-2016).

Epoch	Rmse	Mse	Pearson	Spearman	CI
100	1.4491	2.0998	0.7593	0.7574	0.7826
200	1.4500	2.1024	0.7508	0.7577	0.7857
300	1.4268	2.0357	0.7605	0.7615	0.7866
400	1.3874	1.9250	0.7699	0.7692	0.7902
500	1.4299	2.0447	0.7534	0.7542	0.7815
600	1.4279	2.0390	0.7539	0.7525	0.7807
700	1.4240	2.0279	0.7588	0.7570	0.7848
800	1.3969	1.9514	0.7696	0.7593	0.7859
900	1.4007	1.9620	0.7655	0.7618	0.7849
1000	1.3973	1.9524	0.7659	0.7530	0.7810
1100	1.3867	1.9230	0.7703	0.7642	0.7855
1200	1.3843	1.9162	0.7719	0.7672	0.7891
1300	1.3882	1.9270	0.7696	0.7573	0.7847
1400	1.3981	1.9547	0.7656	0.7533	0.7833
1500	1.4094	1.9864	0.7610	0.7440	0.7768
1600	1.4304	2.0461	0.7533	0.7405	0.7757
1700	1.4292	2.0426	0.7538	0.7407	0.7772
1800	1.4322	2.0511	0.7520	0.7369	0.7750
1900	1.3985	1.9558	0.7694	0.7538	0.7814
2000	1.4190	2.0136	0.7584	0.7431	0.7773

Table S7. The performance of DeepBindGCN_RG_x with different training epoch on the PDBbind v.2013 core set.

Epoch	Rmse	Mse	Pearson	Spearman	CI
100	1.6113	2.5963	0.7078	0.7019	0.7563
200	1.5519	2.4083	0.7340	0.7259	0.7677
300	1.5121	2.2864	0.7415	0.7216	0.7686
400	1.4921	2.2263	0.7493	0.7372	0.7722
500	1.5385	2.3668	0.7295	0.7166	0.7640
600	1.5103	2.2811	0.7413	0.7306	0.7730
700	1.5064	2.2693	0.7460	0.7366	0.7756
800	1.4931	2.2294	0.7506	0.7289	0.7726
900	1.4828	2.1986	0.7514	0.7321	0.7741
1000	1.4823	2.1971	0.7524	0.7339	0.7734
1100	1.4750	2.1756	0.7548	0.7417	0.7758
1200	1.4864	2.2094	0.7503	0.7358	0.7740
1300	1.5318	2.3463	0.7314	0.7153	0.7667
1400	1.4775	2.1830	0.7539	0.7390	0.7779
1500	1.5091	2.2775	0.7414	0.7222	0.7669
1600	1.5226	2.3183	0.7367	0.7219	0.7665
1700	1.5351	2.3564	0.7307	0.7170	0.7659
1800	1.5011	2.2534	0.7450	0.7224	0.7687
1900	1.4844	2.2035	0.7540	0.7375	0.7743
2000	1.4986	2.2458	0.7467	0.7272	0.7725

Table S8. Performance of DeepBindGCN_BC_onehot representation on test set during the training with epoch interval 100.

Epoch	AUC	TPR	Precision	Accuracy
100	0.8420	0.6208	0.5950	0.7996
200	0.8509	0.6088	0.6185	0.8083
300	0.8586	0.6238	0.6204	0.8105
400	0.8615	0.6017	0.6452	0.8177
500	0.8607	0.6075	0.6353	0.8147
600	0.8662	0.6383	0.6289	0.8154
700	0.8684	0.6021	0.6653	0.8248
800	0.8663	0.6263	0.6555	0.8243
900	0.8672	0.6575	0.6345	0.8197
1000	0.8722	0.6208	0.6709	0.8291
1100	0.8722	0.6363	0.6628	0.8281
1200	0.8740	0.6342	0.6708	0.8307
1300	0.8748	0.6254	0.6692	0.8291
1400	0.8758	0.6388	0.6721	0.8318
1500	0.8704	0.6038	0.6806	0.8301
1600	0.8697	0.6442	0.6526	0.8253
1700	0.8719	0.6400	0.6615	0.8281
1800	0.8744	0.6421	0.6668	0.8303
1900	0.8762	0.6746	0.6489	0.8274
2000	0.8667	0.6471	0.6642	0.8300

Table S9. Performance of DeepBindGCN_RG_onehot on test set during the training with epoch interval 100.

Epoch	Rmse	Mse	Pearson	Spearman	CI
100	1.4180	2.0108	0.6785	0.6717	0.7438
200	1.5076	2.2728	0.6620	0.6531	0.7365
300	1.4459	2.0907	0.6772	0.6663	0.7416
400	1.4240	2.0278	0.6813	0.6703	0.7433
500	1.3913	1.9357	0.6904	0.6798	0.7475
600	1.3880	1.9266	0.6920	0.6789	0.7474
700	1.3441	1.8067	0.7066	0.6954	0.7548
800	1.3422	1.8015	0.7072	0.6941	0.7541
900	1.3364	1.7860	0.7147	0.7052	0.7591
1000	1.3249	1.7553	0.7146	0.7025	0.7584
1100	1.3130	1.7241	0.7238	0.7123	0.7625
1200	1.3006	1.6915	0.7249	0.7125	0.7635
1300	1.3008	1.6920	0.7246	0.7129	0.7634
1400	1.3153	1.7299	0.7179	0.7031	0.7589
1500	1.3075	1.7095	0.7226	0.7072	0.7613
1600	1.3154	1.7303	0.7155	0.6988	0.7574
1700	1.3076	1.7099	0.7196	0.7024	0.7589
1800	1.3051	1.7034	0.7223	0.7070	0.7610
1900	1.2987	1.6865	0.7247	0.7088	0.7613
2000	1.3060	1.7058	0.7231	0.7062	0.7607

References

1. DeLano, W.L. Pymol: An open-source molecular graphics tool. *CCP4 Newsl. Protein Crystallogr.* **2002**.
2. Laio, A.; Gervasio, F.L. Metadynamics: A method to simulate rare events and reconstruct the free energy in biophysics, chemistry and material science. *Reports Prog. Phys.* **2008**, doi:10.1088/0034-4885/71/12/126601.
3. Saleh, N.; Ibrahim, P.; Saladino, G.; Gervasio, F.L.; Clark, T. An Efficient Metadynamics-Based Protocol to Model the Binding Affinity and the Transition State Ensemble of G-Protein-Coupled Receptor Ligands. *J. Chem. Inf. Model.* **2017**, *57*, 1210–1217, doi:10.1021/acs.jcim.6b00772.
4. Zhang, H.; Yang, Y.; Li, J.; Wang, M.; Saravanan, K.M.; Wei, J.; Tze-Yang Ng, J.; Tofazzal Hossain, M.; Liu, M.; Zhang, H.; et al. A novel virtual screening procedure identifies Pralatrexate as inhibitor of SARS-CoV-2 RdRp and it reduces viral replication in vitro. *PLoS Comput. Biol.* **2020**, *16*, e1008489, doi:10.1371/journal.pcbi.1008489.
5. Ruiz-Carmona, S.; Schmidtke, P.; Luque, F.J.; Baker, L.; Matassova, N.; Davis, B.; Roughley, S.; Murray, J.; Hubbard, R.; Barril, X. Dynamic undocking and the quasi-bound state as tools for drug discovery. *Nat. Chem.* **2017**, *9*, 201–206, doi:10.1038/nchem.2660.
6. Hess, B.; Kutzner, C.; Van Der Spoel, D.; Lindahl, E. GRGMACS 4: Algorithms for highly efficient, load-balanced, and scalable molecular simulation. *J. Chem. Theory Comput.* **2008**, *4*, 435–447, doi:10.1021/ct700301q.
7. Hornak, V.; Simmerling, C. Generation of accurate protein loop conformations through low-barrier molecular dynamics. *Proteins Struct. Funct. Genet.* **2003**, *51*, 577–590, doi:10.1002/prot.10363.
8. Sousa da Silva, A.W.; Vranken, W.F. ACPYPE - AnteChamber PYthon Parser interfacE. *BMC Res. Notes* **2012**, *5*, 367, doi:10.1186/1756-0500-5-367.
9. Wang, J.; Wang, W.; Kollman, P.A.; Case, D.A. Automatic atom type and bond type perception in molecular mechanical calculations. *J. Mol. Graph. Model.* **2006**, *25*, 247–260, doi:10.1016/j.jmgm.2005.12.005.
10. Jorgensen, W.L.; Chandrasekhar, J.; Madura, J.D.; Impey, R.W.; Klein, M.L. Comparison of simple potential functions for simulating liquid water. *J. Chem. Phys.* **1983**, *79*, 926–935, doi:10.1063/1.445869.
11. Van Der Spoel, D.; Lindahl, E.; Hess, B.; Groenhof, G.; Mark, A.E.; Berendsen, H.J.C. GROMACS: Fast, flexible, and free. *J. Comput. Chem.* 2005, *26*, 1701–1718.
12. Darden, T.; York, D.; Pedersen, L. Particle mesh Ewald: An N·log(N) method for Ewald sums in large systems. *J. Chem. Phys.* **1993**, *98*, 10089–10092, doi:10.1063/1.464397.
13. Hess, B.; Bekker, H.; Berendsen, H.J.C.; Fraaije, J.G.E.M. LINCS: A Linear Constraint Solver for molecular simulations. *J. Comput. Chem.* **1997**, *18*, 1463–1472, doi:10.1002/(SICI)1096-987X(199709)18:12<1463::AID-JCC4>3.0.CO;2-H.
14. Tribello, G.A.; Bonomi, M.; Branduardi, D.; Camilloni, C.; Bussi, G. PLUMED 2: New feathers for an old bird. *Comput. Phys. Commun.* **2014**, *185*, 604–613, doi:10.1016/j.cpc.2013.09.018.
15. Williams, T.; Kelley, C.; Campbell, J.; Cunningham, R.; Denholm, D.; Elber, G.; Fearick, R.; Grammes, C.; Hart, L.; Hecking, L.; et al. Gnuplot 4.6. *Softw. Man.* **2012**.
